# Studies in the rearrangement reactions involving camphorquinone†

**DOI:** 10.1039/d0ra09839f

**Published:** 2021-02-11

**Authors:** H. Surya Prakash Rao, Ahana Saha, Satish Vijjapu

**Affiliations:** Department of Chemistry, Pondicherry University Puducherry 605 014 India profhspr@gmail.com hsprlab@gmail.com +91-9443264222; Syntho-Cascade Research Laboratories Plot No: 65A &B, Survey No: 125, IDA Mallapur, Nacharam Hyderabad-500076 India

## Abstract

Skeletal rearrangements of camphor are well-known, however, those involving camphorquinone, its sibling, are rare. We have found that the diol derived from allylated camphorquinone undergoes iodine or bromine mediated deep-seated skeletal rearrangement to provide an interesting tricyclic ring system. The iodo group in the rearranged product provided convenient leverage for further functionalization. For example, it was converted into an azide and the azide was subjected to copper(i) mediated Huisgen 1,3-dipolar cycloaddition with acetylenes to obtain a terpene–triazole conjugate.

## Abstract 2

We described an unprecedented skeletal rearrangement of camphorquinone motif to an interesting tricyclic ring system.Peripheral functional group modification provided a terpene-triazole conjugate.

## Graphical abstract

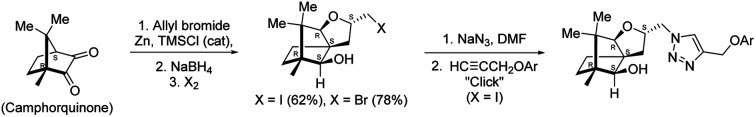

## 1 Introduction

**Fig. 1:**
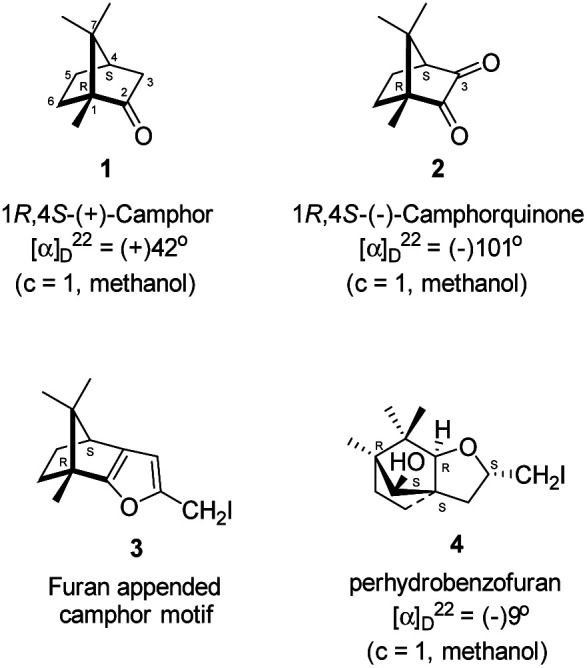
Structures of (+)-camphor 1 and (−)-camphorquinone 2, furan appended camphor motif 3 intended for preparation and perhydrobenzofuran 4 actually formed.

Camphor (Sanskrit, Korpur) 1 (Fig. 1), a quintessential natural product, has been known to humankind since ancient times.^1–3^ It is widely distributed in many plant species, specifically abundant in the essential oils extracted from the woody parts of the Borneo camphor tree (*Dryobalanops aromatica*), the East African camphor wood tree (*Ocoteaus ambarensis*), Chinese camphor tree (*Cinnamomum camphora*), Greek sage (*Salvia fruticosa*), Spanish sage (*Salvia lavandulifolia*), Lavender cotton (*Santolina insularis*) *etc.*^4^ It is a classic example of a monoterpene. The extraordinarily compact structure of camphor, forced to be in an uncomfortable boat form of cyclohexane, has been known to go through extraordinarily diverse skeletal rearrangements and ring opening reactions.^5^ Over a century back, Lowry and Armstrong noted, “No substance known to us suffers rearrangement of its parts and undergoes a complete change of type more readily than does camphor”.^6^ Relief of the strain and the anti-periplanar nature of the C–C bonds in the norbornane ring system are driving forces for the rearrangement reactions, possibly *via* the intermediates involving non-classical carbocations. The chemistry of skeletal rearrangements of camphor has been a topic of interest throughout the history of natural product chemistry. Rearrangement reactions involving camphorquinone^7^2 (Fig. 1), its sibling, however, did not receive much attention. Camphorquinone 2 is commercially available and is used as a photo-initiator in dentistry.^8^ It can be prepared readily, in high yield by oxidation of camphor 1 with selenium dioxide^9–12^ or phenylselenous acid anhydride^13^ or by aerial oxidation of 3-bromocamphor in the presence of sodium iodide and DMSO.^14–16^

Camphorquinone, 2, like camphor 1, is embodied with two well defined stereogenic carbons. It, however, is more strained and more reactive than camphor 1, primarily due to the presence of one more sp^2^ carbon C(3) in the ring. Indeed, two carbonyl groups located on C(2) and C(3) of 2 can be differentiated readily in nucleophilic reactions. Due to CMe at C(1), adjacent C(2) carbonyl group is more sterically hindered to approach of the nucleophiles than to C(3) carbonyl.^17–19^ Furthermore, reactions at C(3) carbonyl group with nucleophiles exhibit high stereo-selectivity due to steric hindrance from the gem-methyl groups located on C(7). High regio-, chemo- and stereo-selectivity in the reactions with nucleophiles and potential for rearrangements make it an attractive starting compound for synthetic endeavours. Surprisingly however, it did not find many applications in organic synthesis.^20^ Previously, we have established a method for the synthesis of 2-methylfuran appended polycyclic aromatic hydrocarbons (PAHs) from corresponding 1,2-diketones.^21^ Barbier type allylation on one of the two carbonyl groups, reduction of the remaining carbonyl, iodine mediated 5-*exo*-dig cyclization yielded 5-methylfuran appended to PAHs. Since camphorquinone 2 has 1,2-diketone functionality, we planned to synthesize 3 (Fig. 1) where furan is appended to camphor motif. We thought that, similar to 2, furan appended strained molecules like 3 could exhibit interesting photochemical properties. In this effort, to our pleasant surprise, instead of observing formation of 3 in the iodine mediated cyclization step, we unearthed a deep-seated rearrangement of the camphor skeleton to form perhydrobenzofuran 4 (Fig. 1).

## 2 Results and discussion

**Scheme 1:**
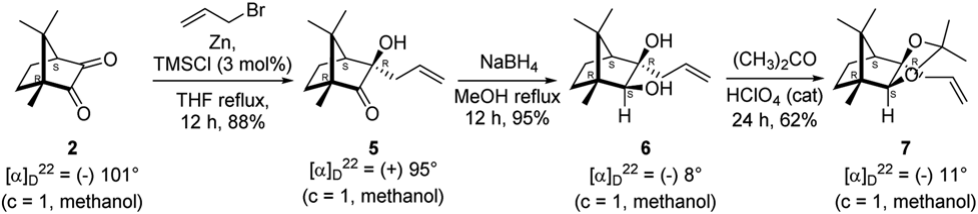
Synthesis of camphor derived 1,2-diol 6 and its acetonoide 7.

The Barbier type reaction of 1*R*,4*S*-(−)-camphorquinone 2 with allyl bromide in presence of zinc metal and trimethylsilyl chloride (TMSCl, 3 mol%) resulted in regio- (at C(3) out of the possible C(2) and C(3)) and stereo- (*endo*- out of possible *exo*- and *endo*-) selective allylated product 5 in 88% yield (Scheme 1). The reagent TMSCl was used for initial activation of zinc.^22^ Allylation took place with equal facility in presence of indium, but, as zinc is relatively inexpensive and easily available, we used it. The allylation took place with equal facility even when the reaction was conducted on 10 mmol of 2. Reduction of the C(2) carbonyl group in 5 with sodium borohydride in methanol reflux provided *exo*, *exo*-diol 6 (*exo*- out of possible *exo*- and *endo*-) exclusively in near quantitative yield. In the ^1^H NMR spectrum of the diol 6, C(2)H appeared at *δ* 3.2 ppm which inferred its *endo* orientation.^23^ Stereochemistry of the vicinal diol 6 was further confirmed by its facile conversion to acetonoide 7.

**Scheme 2:**
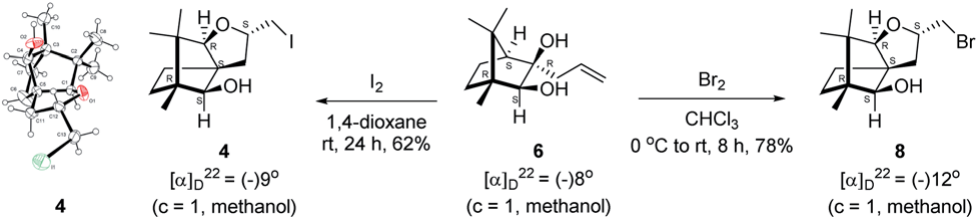
I_2_ and Br_2_ induced skeletal rearrangement of vicinal diol 6 to form 4 and 8 respectively and ORTEP diagram of 4.

We conducted the iodine mediated cyclization of 6 in dioxane medium and the reactions yielded tricyclic product 4 in 62% yield as the only isolable product (Scheme 2). The reaction did not provide even a trace amount of the anticipated furan 3 (Scheme 1). In the ^1^H NMR spectrum of 4, three singlet signals at *δ* 1.08, 0.98 and 0.96 ppm, for methyl groups, which when compared to the corresponding signals for the diol 6 (*δ* 1.18, 0.91, 0.84 ppm) were distinctly different. The ^1^H decoupled ^13^C NMR spectrum of 4 revealed 13 signals, among which signals at 96.7, 89.0 and 81.0 ppm were of oxygen-attached CH type (from DEPT spectra). The vicinal diol 6, on the other hand, exhibited two oxygen-attached-carbon signals located at 85.3 (CH) for C(2) and 78.0 (C) for C(3). Since both ^1^H and ^13^C NMR spectra of 4 were distinctly different from what is anticipated from the camphor framework, we concluded that there has been a deep-seated rearrangement of the skeleton. The structure of 4 was further delineated from the analysis of 2D NMR (COSY, HSQC and HMBC) spectral data. Finally, we confirmed the assigned structure 4 by the analysis of the single crystal X-ray crystallographic data (Scheme 2).^23^ The tricyclic product 4 has interesting structural features like spirobicyclic system and perhydrobenzofuran motif. The reaction of 6 with Br_2_ in CHCl_3_ proceeded along similar path to provide perhydrobenzofuran 8 in 78% yield. The both ^1^H and ^13^C NMR spectra of 8 compared well with those of 4.

**Scheme 3:**
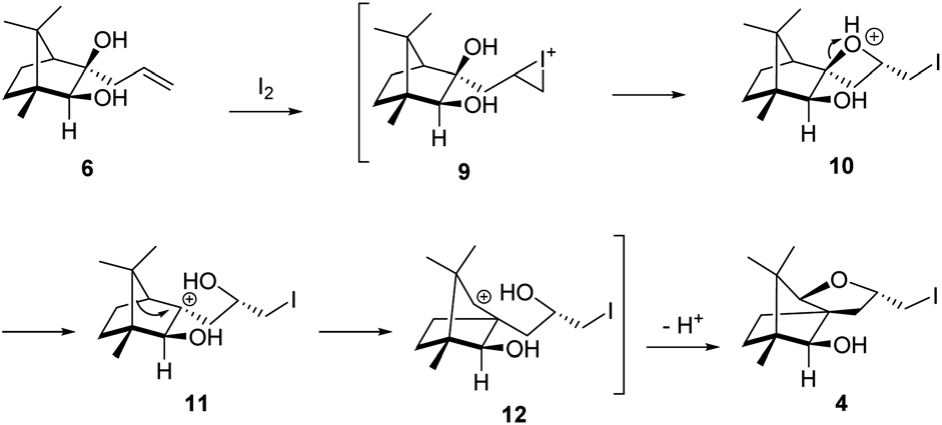
Plausible mechanistic course for the iodine induced rearrangement of 6 to 4.

The plausible mechanistic course for skeletal rearrangement of camphor motif in 6 is given in Scheme 3. Electrophilic addition of iodine to terminal double bond in 6 leads to iodonium ion intermediate 9. Stereospecific ring opening of three membered cyclic iodonium ion 9 with C(3) hydroxyl group (path A) instead of C(2) hydroxyl group (path B) lead to formation of the spirocyclic oxetane 10. If C(2) hydroxyl was involved in the reaction, it would have provided the furan derivative 3. We think that proximity of hydroxyl group at C(3) position was responsible for the reaction to go through path A. Strain and proton induced ring opening of four-membered ring in 10 leads to carbocation 11. Concomitant migration of the anti-periplanar bond as seen in 10 generates carbocation 12. Quenching of the secondary carbocation in 12 with proximate hydroxyl group leads to final product 4. Similar to skeletal rearrangements in terpenoids, there may not be well-defined intermediates like 10, 11 and 12 in the pathway. Indeed, single product 4 obtained in the reaction indicates that the rearrangement may not be step-wise as shown in Scheme 3.

**Scheme 4:**
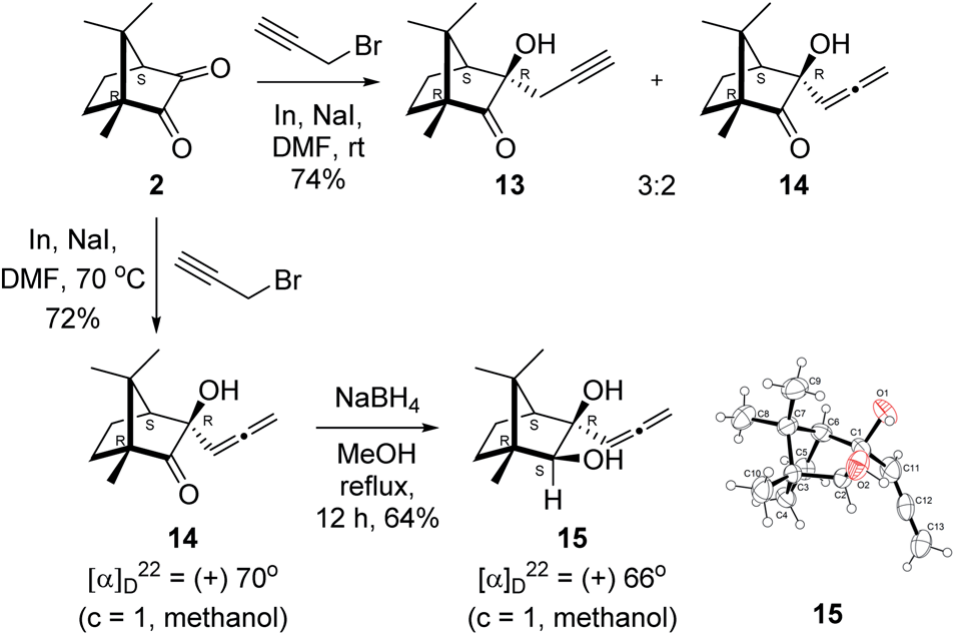
Propargylation/allenylation of camphorquinone 2 to 13 and 14. Reduction 14 to 15 and ORTEP diagram of 15.

Next, we studied propargylation of camphorquinone 2 with propargyl bromide. The indium or zinc metal mediated Barbier type propargylation of (1*R*,4*S*)-(−)-camphorquinone 2 resulted in inseparable mixture of two isomeric *tert*-α-hydroxy ketones, namely the propargylated 13 and the allenylated 14 products in 6 : 4 ratios in 74% yield (Scheme 4). Of the two, the reaction with indium was cleaner. Optimization of the reaction conditions towards single product resulted in finding that when the indium mediated reaction was carried out at 70 °C in DMF medium in presence of 1 equivalent of NaI, the reaction provided single regio-selective and stereo-selective allenylated product 14 in 72% yield. Stereo-selective reduction of the keto group in 14 with sodium borohydride in methanol reflux provided 1,2-diol 15 (Scheme 4). Structure of the diol 15 was confirmed by ^1^H NMR, ^13^C NMR, DEPT-135 spectra and furthermore unambiguously confirmed by single crystal X-ray structural analysis (Scheme 4).^23^ Unfortunately, our attempts to induce iodine or bromine mediated deep-seated rearrangement of camphor skeleton in 15 as found in the case of 6 did not yield tangible results.

**Scheme 5:**
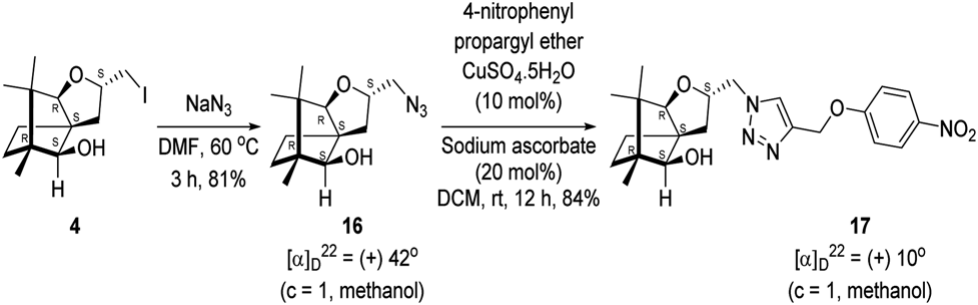
Synthesis of the 1,4-disubstituted triazole 17 from 4*via* the azide 16.

Next, we sought to elaborate 4 into further complex products. In this quest, we planned substitution of iodo group with azide so that 1,3-dipolar cycloaddition with terminal acetylenes are possible. The reaction of 4 with sodium azide in dimethylformamide (DMF) led to the corresponding azide 16 in 81% yield (Scheme 5). As a representative example, the azide was subjected to Husigen's 1,3-dipolar cycloaddition with 4-nitrophenyl propargyl ether in presence of copper sulfate pentahydrate and sodium ascorbate (Click reaction)^24^ in dichloromethane. The reaction provided anticipated 1,4-disubstituted triazole 17 in 84% yield. Structure of 17 was confirmed by analysis of spectroscopic (IR, ^1^H, ^13^C, DEPT) and HRMS data.

## 3 Conclusions

In summary, we have shown that the allylated diol 6, derived from camphorquinone 2 in chemo-, regio- and stereo-selective manner in two simple steps, undergoes a deep-seated rearrangement when treated with iodine or bromine to provide 4/8 which possess interesting and unprecedented tricyclic ring system. The reaction of 2 with propargyl bromide and indium resulted in allenylated product but it failed to undergo rearrangement reaction. The functional group transformation of the iodo group in 4 into azide 16 followed by copper(i) mediated Huisgen 1,3-dipolar cycloaddition with an acetylene furnished 1,4-disubstituted triazole 17. We anticipate that present findings will lead to further exploration in the rearrangement reactions involving camphorquinone and attachment of such rearranged products to biomolecules.

## 4 Experimental section

### 4.1. General experimental procedure

All reactions were carried out in oven dried glass-wares. Solvents were dried according to the standard procedures given in literature. Thin layer chromatography (TLC) was used for monitoring the reactions. Silica gel G and GF 254 coated glass plates were used for making TLC plates. Purification was done by using column chromatography technique by using 100–200 mesh silica from Avra Synthesis Pvt. Ltd and hexanes and ethyl acetate. Melting points were uncorrected and were recorded on VEEGO VMP-DS instrument by using open-ended capillary tubes. IR spectra were recorded as KBr pellets on a Nicolet-6700 spectrometer. NMR (^1^H, ^13^C and DEPT-135) spectra were recorded by using Bruker Avance 400 spectrometer with tetramethylsilane (TMS) as the internal standard and (CDCl_3_ and CDCl_3_ + CCl_4_, 1 : 1) solutions; *J*-values are in Hz. ^1^H-NMR data are reported as follows: chemical shift (multiplicity (s = singlet, d = doublet, t = triplet, q = quartet, m = multiplet, dd = doublet of doublet and br s = broad singlet), coupling constant (*J*) and integrations). High resolution mass spectra (HRMS) were recorded on Agilent 6350 B Q-TOF mass spectrometer using electro spray ionization mode.

The optical activity of all the compounds were recorded in AUTOPOL-IV Automatic Polarimeter where the D-line of sodium was used as the polarized source of light.

### 4.2. Allylation of (1*R*,4*S*)-(−)-camphorquinone 2


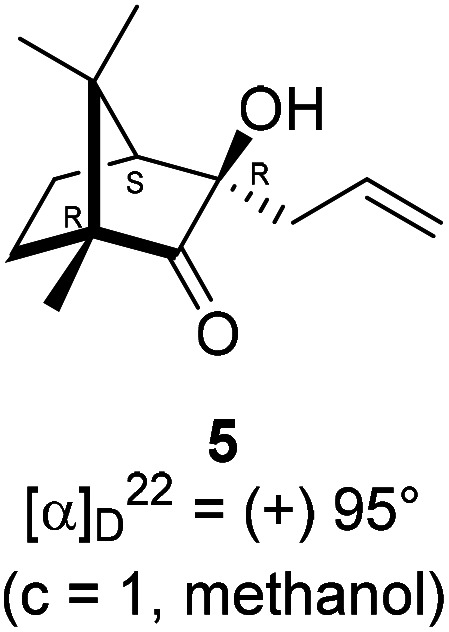
In a two necked 100 mL round bottom flask, zinc (78.8 mg, 1.21 mmol) was suspended in THF (8 mL). To this suspension, a solution of TMSCl (3 mol%) in xylene (1 mL) was added. Resulting mixture was heated to reflux along with vigorous stirring for 30 min. To the resulting suspension, (1*R*,4*S*)-(−)-camphorquinone 2 (200 mg, 1.21 mmol) in THF (5 mL) and allyl bromide (218.7 mg, 1.807 mmol) in THF (5 mL) were added drop wise simultaneously by using two syringes for a period of 30 min. After addition, the reaction mixture which was orange in color was heated at 45 °C till all the camphorquinone had been consumed and the color changed to light yellow. After completion of the reaction, (as monitored by TLC, *R*_f_ = 0.22, (10% EtOAc/hexane)) the crude product was extracted using dichloromethane (DCM, 30 mL). The DCM layer was washed with water (2 × 30 mL), brine solution (2 × 30 mL) and then dried over anhydrous sodium sulfate. The concentrated crude product was purified by column chromatography using silica gel (100–200 mesh) with increasing amounts of EtOAc (2 to 4%) in hexane to afford (1*R*,3*R*,4*S*)-(+)-3-allyl-3-hydroxy-1,7,7-trimethylbicyclo[2.2.1]heptan-2-one 5 as a light yellow colored semi-solid (220 mg, 88% yield). [*α*]^22^_D_ = +95° (*c* = 1, MeOH); IR (KBr, cm^−1^) 3479, 3077, 2959, 2874, 1747, 1639, 1494, 1455, 1394, 1293, 1220, 1105, 998, 953, 916, 861, 750; ^1^H NMR (400 MHz, CDCl_3_ + CCl_4_, 1 : 1) *δ* 5.92–5.81 (m, 1H), 5.13–5.09 (m, 2H), 2.75 (s, 1H), 2.27 (dd, *J* = 14.8, 6.6 Hz, 1H), 2.19 (dd, *J* = 14.8, 7.7 Hz, 1H), 1.92 (d, *J* = 4.2 Hz, 1H), 1.85–1.35 (m, 4H), 0.98 (s, 3H), 0.92 (s, 3H), 0.85 (s, 3H);^13^C NMR (100 MHz, CDCl_3_ + CCl_4_, 1 : 1) *δ* 220.3 (C), 132.6 (CH),119.6 (CH_2_), 77.8 (C), 58.5 (C), 51.8 (CH), 46.4 (C), 41.1 (CH_2_), 30.0 (CH_2_), 22.6 (CH_3_), 22.4 (CH_2_), 20.6 (CH_3_), 9.7 (CH_3_); HRMS (ESI) calcd for C_13_H_21_O_2_ [M + H]^+^ 209.1542, found 209.1524.

### 4.3. Reduction of keto alcohol 5 to diol 6


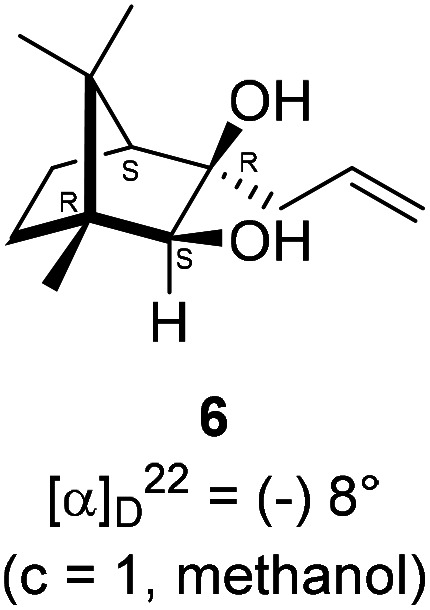
In a single necked 100 mL round bottom flask, (1*R*,3*R*,4*S*)-(+)-3-allyl-3-hydroxy-1,7,7-trimethylbicyclo[2.2.1]heptan-2-one 5 (50 mg, 0.240 mmol) was dissolved in MeOH (4 mL), and sodium borohydride (18.2 mg, 0.48 mmol) was added in portions slowly at room temperature and refluxed for 12 h. After completion of the reaction (by TLC, *R*_f_ = 0.43, (10% EtOAc/hexane)), MeOH was evaporated. Then the crude product was extracted using dichloromethane (10 mL). The DCM layer was then washed with water (2 × 10 mL), brine solution (2 × 10 mL) and then dried over anhydrous sodium sulfate. Then concentrated crude product was purified by column chromatography using silica gel (100–200 mesh) and increasing amounts of (5 to 8%) EtOAc in hexanes led to (1*R*,2*S*,3*R*,4*S*)-(−)-3-allyl-1,7,7-trimethylbicyclo[2.2.1]heptane-2,3-diol 6 as white semisolid (48 mg, 95% yield); [*α*]^22^_D_ = −8°(*c* = 1, MeOH); IR (KBr, cm^−1^) 3479, 3077, 2959, 2874, 1747, 1693, 1494, 1455, 1394, 1293, 1220, 1154, 1044, 998, 953, 916, 861, 750, 691, 616, 562; ^1^H NMR (400 MHz, CDCl_3_ + CCl_4_, 1 : 1) *δ* 5.89 (ddt, *J* = 17.8, 10.7, 7.3 Hz, 1H), 5.18 (dd, *J* = 13.3, 4.8 Hz, 2H), 3.19 (d, *J* = 5.9 Hz, 1H), 2.59–2.58 (m, 1H), 2.36 (td, *J* = 14.0, 7.2 Hz, 3H), 1.68 (d, *J* = 4.2 Hz, 1H), 1.53 (dd, *J* = 30.3, 8.0 Hz, 3H), 1.38–1.30 (m, 1H), 1.18 (s, 3H), 0.91 (s, 3H), 0.84 (s, 3H);^13^C NMR (100 MHz, CDCl_3_ + CCl_4_, 1 : 1) *δ* 134.3 (CH), 118.5 (CH_2_), 85.5 (CH), 79.0 (C), 54.1 (CH), 49.7 (C), 48.7 (C), 44.9 (CH_2_), 33.7 (CH_2_), 22.6 (CH_3_), 22.4 (CH_3_), 22.1 (CH_2_), 11.6 (CH_3_); HRMS (ESI) calcd for C_13_H_23_O_2_ [M + H]^+^ 211.1698, found 211.1694.

### 4.4. Synthesis of acetonide 7 of diol 6


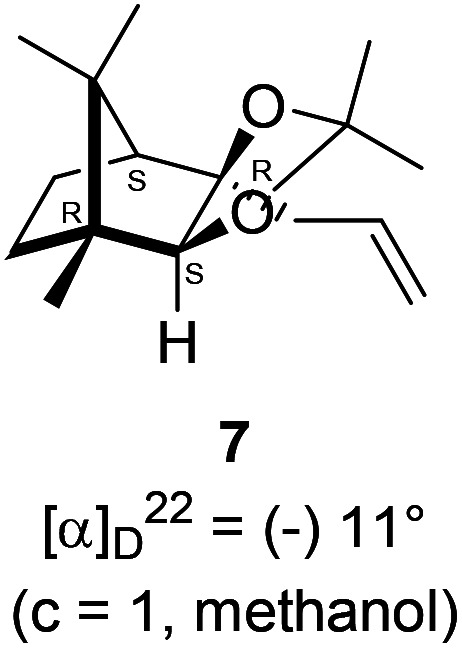
In a 100 mL single necked round bottom flask, (1*R*,2*S*,3*R*,4*S*)-(−)-3-allyl-1,7,7-trimethylbicyclo[2.2.1]heptane-2,3-diol 6 (50 mg, 0.238 mmol) was dissolved in distilled acetone (5 mL) and was treated with 70% aqueous perchloric acid (0.2 mL) at room temperature and stirred for 24 h. After completion of the reaction (confirmed by TLC, *R*_f_ = 0.47, (10% EtOAc/hexane)), reaction was stopped by addition of excess solid NaHCO_3_ and the organic layer was collected separately. The solvent acetone was evaporated and the concentrated crude product so obtained, was purified by column chromatography using silica (100–200 mesh) and increasing amount of 5 to 10% EtOAc in hexanes afforded (3a*R*,4*S*,7*R*,7a*S*)-(−)-3a-allyl-2,2,7,8,8-pentamethylhexahydro-4,7-methanobenzo[*d*][1,3]dioxole 7 as a yellow semi solid (40 mg, 62% yield); [*α*]^22^_D_ = −11° (*c* = 1, MeOH); IR (KBr, cm^−1^) 2930, 2870, 1736, 1645, 1455, 1374, 1260, 1192, 1035, 998, 909, 803, 661; ^1^H NMR (400 MHz, CDCl_3_ + CCl_4_, 1 : 1) *δ* 5.98–5.88 (m, 1H), 5.10–5.06 (m, 2H), 3.54 (s, 1H), 2.63–2.62 (m, 1H), 2.46–2.40 (m, 1H), 2.30–2.22 (m, 2H), 1.91 (d, *J* = 4.3 Hz, 1H), 1.61–1.57 (m, 1H), 1.55 (s, 3H), 1.43 (s, 3H), 1.30–1.26 (m, 1H), 1.22 (s, 3H), 0.98 (s, 3H), 0.84 (s, 3H); ^13^C NMR (100 MHz, CDCl_3_ + CCl_4_) *δ* 134.6 (CH), 117.4 (CH_2_), 108.8 (C),91.9 (CH), 90.2 (C), 51.4 (CH), 48.8 (C), 47.4 (C), 42.8 (CH_2_), 31.9 (CH_2_), 27.8 (CH_3_), 26.6 (CH_3_), 24.3 (CH_3_), 22.6 (CH_2_), 20.9 (CH_3_), 12.1 (CH_3_); HRMS (ESI) calcd for C_16_H_27_O_2_ [M + H]^+^ 250.1933, found 250.1998.

### 4.5. Iodine mediated rearrangement of 6 to provide the iodide 4


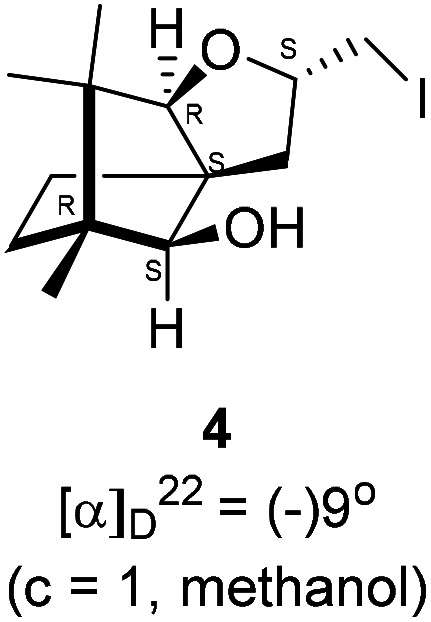
In a 100 mL single necked round bottom flask, (1*R*,2*S*,3*R*,4*S*)-(−)3-allyl-1,7,7-trimethylbicyclo[2.2.1]heptane-2,3-diol 6 (50.4 mg, 0.24 mmol) was dissolved in 1,4-dioxane (2 mL) and then iodine (93.2 mg, 0.36 mmol), was added to it and stirred at room temperature for 24 h. After completion of reaction (monitored by TLC, *R*_f_ = 0.44 (10% EtOAc/hexane)) the solvent was evaporated and the crude product was extracted using dichloromethane (20 mL). The organic layer was washed with hypo solution (2 × 20 mL), brine solution (2 × 20 mL) and then dried over anhydrous sodium sulfate. The concentrated crude product was purified by column chromatography using silica (100–200 mesh) and 9% EtOAc/hexane as eluent to afford (2*S*,3a*R*,6*S*,7a*R*,8*S*)-(−)-2-(iodomethyl)-6,7,7-trimethylhexahydro-2*H*-3a,6-methanobenzofuran-8-ol 4 as a white semi solid (70.2 mg, 88% yield); [*α*]^22^_D_ = −9° (*c* = 1, MeOH); IR (KBr, cm^−1^) 3466, 3418, 2955, 2926, 2868, 1669, 1598, 1453, 1380, 1132, 1034, 995, 761, 701, 622; ^1^H NMR (400 MHz, CDCl_3_ + CCl_4_, 1 : 1) *δ* 4.48–4.41 (m, 1H), 3.56 (d, *J* = 1.8 Hz, 1H), 3.29–3.26 (m, 2H), 3.12 (t, *J* = 9.1 Hz, 1H), 2.38 (d, *J* = 10.7 Hz, 1H), 2.39–2.34 (m, 1H), 1.68–1.65 (m, 3H), 1.27–1.14 (m, 2H), 1.08 (s, 3H), 0.97 (s, 3H), 0.96 (s, 3H); ^13^C NMR (400 MHz, CDCl_3_ + CCl_4_, 1 : 1) *δ* 96.7 (CH), 89.0 (CH), 81.0 (CH), 57.4 (C), 50.8 (C), 43.4 (C), 35.2 (CH_2_), 29.8 (CH_2_), 26.8 (CH_3_), 23.4 (CH_2_), 21.4 (CH_3_), 13.5 (CH_3_), 11.4 (CH_2_); HRMS (ESI) calcd for C_13_H_22_IO_2_ [M + H]^+^ 337.0664, found 337.0659.

### 4.6. Bromine mediated rearrangement of 6 to provide the bromide 8


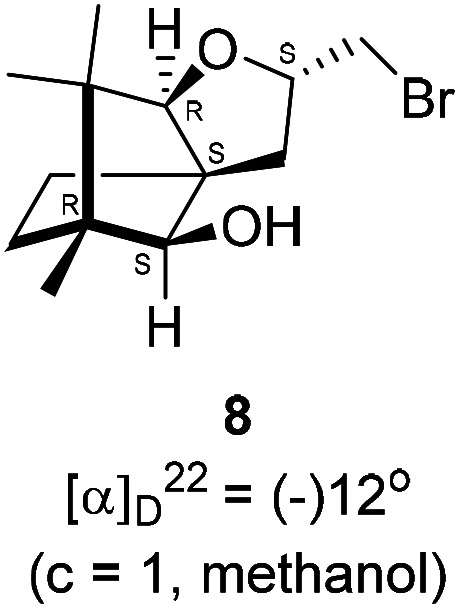
In a 100 mL single necked round bottom flask, (1*R*,2*S*,3*R*,4*S*)-(−)-3-allyl-1,7,7-trimethylbicyclo[2.2.1]heptane-2,3-diol 6 (50 mg, 0.238 mmol) was dissolved in distilled CHCl_3_ (3 mL). The solution was kept in ice-bath, so that the temperature was maintained below 0 °C. Then, bromine (45.70 mg, 0.286 mmol) in CHCl_3_ solution (2 mL) was added to it drop-wise, and the reaction mixture was stirred at room temperature. After completion of the reaction after 8 h (as confirmed by TLC, *R*_f_ = 0.21, (8% EtOAc/hexane)), the crude product was extracted using DCM (10 mL). The DCM layer was washed with water (2 × 10 mL), brine solution (2 × 10 mL) and then dried over anhydrous sodium sulfate. Then concentrated crude product was purified by column chromatography using silica gel (100–200 mesh) and increasing amounts of (5 to 8%) EtOAc in hexanes furnished (2*S*,3a*R*,6*S*,7a*R*,8*S*)-(−)-2-(bromomethyl)-6,7,7-trimethylhexahydro-2*H*-3a,6-methanobenzofuran-8-ol 8 as yellowish liquid (53 mg, 78% yield). [*α*]^22^_D_ = −12° (*c* = 1, MeOH); IR (KBr, cm^−1^) 3435, 2951, 2884, 2726, 1451, 1384, 1281, 1233, 1124, 1092, 1047, 833, 657, 493; ^1^H NMR (400 MHz, CDCl_3_ + CCl_4_, 1 : 1) *δ* 3.81 (d, *J* = 7.0 Hz, 1H), 3.58 (d, *J* = 6.9 Hz, 1H), 2.88 (s, 2H), 1.75 (d, *J* = 4.8 Hz, 1H), 1.72–1.68 (m, 1H), 1.64 (dd, *J* = 8.1, 4.7 Hz, 1H), 1.46 (dd, *J* = 19.6, 10.9 Hz, 1H), 1.25 (s, 1H), 1.09 (s, 3H), 0.96 (s, 2H), 0.93 (s, 3H), 0.80 (s, 3H); ^13^C NMR (100 MHz, CDCl_3_ + CCl_4_, 1 : 1) *δ* 96.66 (CH), 88.99 (CH), 81.02 (CH), 57.34 (C), 50.77 (C), 43.38 (C), 35.21 (CH_2_), 29.77 (CH_2_), 26.88 (CH_2_), 23.78 (CH_2_), 21.35 (CH_3_), 13.50 (CH_3_), 11.32 (CH_2_); HRMS (ESI) calcd for C_13_H_22_BrO_2_ [M + H]^+^ 289.0703, found 289.0795.

### 4.7. Allenylation of (1*R*,4*S*)-(−)-camphorquinone 2


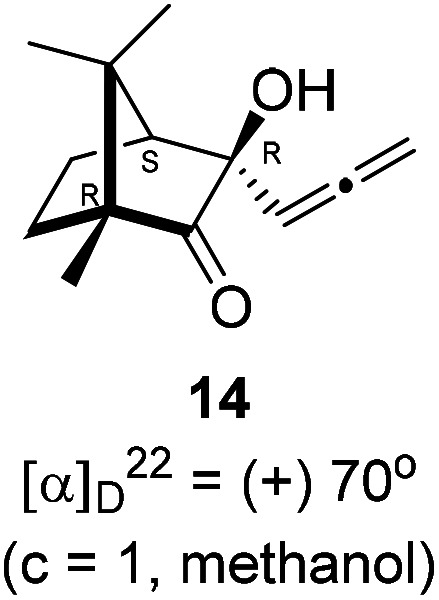
(1*R*,4*S*)-(−)-Camphorquinone 2 (201.3 mg, 1.20 mmol) was dissolved in dimethyl formamide (5 mL) and then 80% propargyl bromide in toluene (277.8 mg, 1.87 mmol), indium metal (138.3 mg, 1.20 mmol), sodium iodide (280.2 mg, 1.87 mmol) were added and stirred at 70 °C for 24 h and then after completion of reaction (by TLC, *R*_f_ = 0.4, (10% EtOAc/hexane)), 1 *N* HCl solution was added to the reaction mixture and the organic layer was extracted using dichloromethane (30 mL) and washed with water (2 × 30 mL), brine solution (2 × 30 mL) and dried over anhydrous sodium sulfate. The concentrated crude product was purified by column chromatography using silica gel (100–200 mesh) and increasing amounts of (2 to 5%) EtOAc in hexanes to afford (1*R*,3*R*,4*S*)-(+)-3-hydroxy-1,7,7-trimethyl-3-(propa-1,2-dien-1-yl)bicyclo[2.2.1]heptan-2-one 14 as a light yellow semi solid (178 mg, 72% yield); [*α*]^22^_D_ = +70° (*c* = 1, MeOH); IR (KBr, cm^−1^) 3385, 2939, 2885, 1956, 1745, 1465, 1382, 1287, 1097, 1052, 849; ^1^H NMR (400 MHz, CDCl_3_ + CCl_4_, 1 : 1) *δ* 5.27 (t, *J* = 6.6 Hz, 1H), 4.85 (ddd, *J* = 27.4, 11.2, 6.7 Hz, 2H), 2.88 (s, 1H), 2.01 (d, *J* = 4.4 Hz, 1H), 1.88–1.32 (m, 4H), 1.04 (s, 3H), 0.96 (s, 3H), 0.88 (s, 3H); ^13^C NMR (100 MHz, CDCl_3_ + CCl_4_, 1 : 1) *δ* 217.7 (C), 206.9 (C), 93.4 (CH), 79.3 (C), 78.4 (CH_2_), 58.3 (C), 52.9 (CH), 46.5 (C), 28.7 (CH_2_), 23.7 (CH_2_), 22.4 (CH_3_), 20.9 (CH_3_), 9.7 (CH_3_); HRMS (ESI) calcd for C_13_H_19_O_2_ [M + H]^+^ 207.1385, found 207.1380.

### 4.8. Synthesis of allenyl diol 15


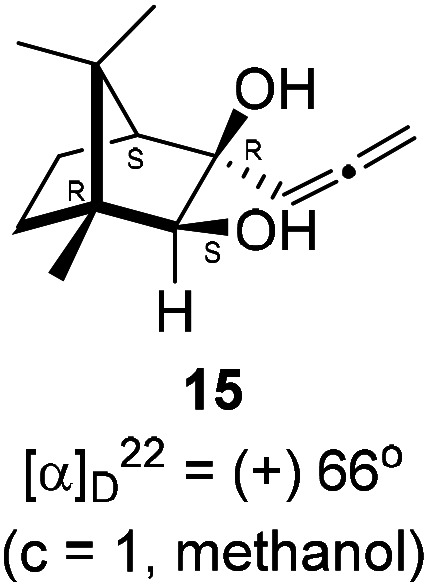
(1*R*,3*R*,4*S*)-(+)-3-Hydroxy-1,7,7-trimethyl-3-(propa-1,2-dien-1-yl)bicyclo[2.2.1]heptan-2-one 14 (501.3 mg, 2.43 mmol) was dissolved in methanol (3 mL) and then sodium borohydride (276.8 mg, 7.3 mmol) was added in portions and refluxed for 12 h and then after completion of the reaction (by TLC, *R*_f_ = 0.26, (10% EtoAc/hexane)) MeOH was evaporated. Then the crude product was extracted using dichloromethane (30 mL). The DCM layer was washed with water (2 × 30 mL), brine solution (2 × 30 mL) and then dried over anhydrous sodium sulfate. Then concentrated crude product was purified by column chromatography using silica gel (100–200 mesh) and increasing amounts of (5 to 10%) EtOAc in hexanes to furnish (1*R*,2*S*,3*R*,4*S*)-(+)-1,7,7-trimethyl-3-(propa-1,2-dien-1-yl)bicyclo[2.2.1]heptane-2,3-diol 15 as a white semi solid (323 mg, 64% yield); mp 62–64 °C; [*α*]^22^_D_ = +66° (*c* = 1, MeOH); IR (KBr, cm^−1^) 3380, 2939, 2885, 1956, 1465, 1382, 1287, 1097, 1052, 849; ^1^H NMR (400 MHz, CDCl_3_ + CCl_4_, 1 : 1) *δ* 5.36 (t, *J* = 6.6 Hz, 1H), 4.83 (d, *J* = 6.7 Hz, 2H), 3.54 (d, *J* = 6.3 Hz, 1H), 3.42 (s, 2H), 3.40 (d, *J* = 6.9 Hz, 1H), 1.69 (d, *J* = 4.0 Hz, 1H), 1.55–1.49 (m, 1H), 1.44–1.37 (m, 2H), 1.19 (s, 3H), 0.92 (s, 3H), 0.85 (s, 3H); ^13^C NMR (100 MHz, CDCl_3_ + CCl_4_, 1 : 1) *δ* 206.2 (C), 97.7 (CH), 82.8 (CH), 79.3 (C), 78.3 (CH_2_), 55.3 (CH), 49.9 (C), 49.1 (C), 33.3 (CH_2_), 22.9 (CH_2_), 22.8 (CH_3_), 22.3 (CH_3_), 11.7 (CH_3_); HRMS (ESI) calcd for C_13_H_20_O_2_H [M + H]^+^ 209.1542, found 209.1534.

### 4.9. Synthesis of the azide 16


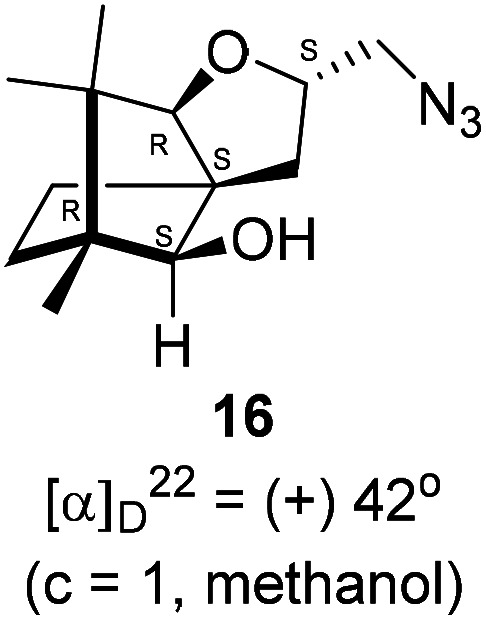
(2*S*,3a*R*,6*S*,7a*R*,8*S*)-(−)-2-(Iodomethyl)-6,7,7-trimethylhexahydro-2*H*-3a,6-methanobenzofuran-8-ol 4 (20.2 mg, 0.06 mmol) was dissolved in dimethylformamide (5.0 mL) and then to it sodium azide (6.1 mg, 0.09 mmol) was added and stirred at 60 °C for 3 h. After the completion of reaction (monitored by TLC, *R*_f_ = 0.25, (10% EtOAc/hexane)), DMF was evaporated. Then the crude product was extracted using DCM (30 mL). The DCM layer was washed with water (2 × 30 mL), brine solution (2 × 30 mL) and then dried over anhydrous sodium sulfate. Then concentrated crude product was purified by column chromatography using silica gel (100–200 mesh) and increasing amount of 4 to 9% EtOAc in hexanes resulted (2*S*,3a*R*,6*S*,7a*R*,8*S*)-(+)-2-(azidomethyl)-6,7,7-trimethylhexahydro-2*H*-3a,6-methanobenzofuran-8-ol 15 as a colorless liquid (12 mg, 81% yield); [*α*]^22^_D_ = +42° (*c* = 1, MeOH); IR (KBr, cm^−1^) 3513, 2927, 2866, 2099, 1457, 1271, 1035, 804, 692, 493; ^1^H NMR (400 MHz, CDCl_3_ + CCl_4_, 1 : 1) *δ* 4.48–4.41 (m, 1H), 3.56 (d, *J* = 1.9 Hz, 1H), 3.29–3.26 (m, 2H), 3.14–3.12 (m, 1H), 2.43 (d, *J* = 10.7 Hz, 1H), 2.36 (dd *J* = 13.2, 7.4 Hz, 1H), 1.68–1.64 (m, 3H), 1.22–1.13 (m, 2H), 1.11 (s, 3H), 0.99 (s, 3H), 0.96 (s, 3H); ^13^C NMR (400 MHz, CDCl_3_ + CCl_4_, 1 : 1) *δ* 96.5 (CH), 89.1 (CH), 80.4 (CH), 56.8 (C), 55.1 (C), 50.8 (C), 43.2 (CH_2_), 31.5 (CH_2_), 29.9 (CH_2_), 26.8 (CH_3_), 23.7 (CH_2_), 21.5 (CH_3_), 13.6 (CH_3_); HRMS (ESI) calcd for C_13_H_22_N_3_O_2_ [M + H]^+^ 252.1706, found 252.1707.

### 4.10. Copper(i) mediated alkyne azide cycloaddition to provide triazole 17


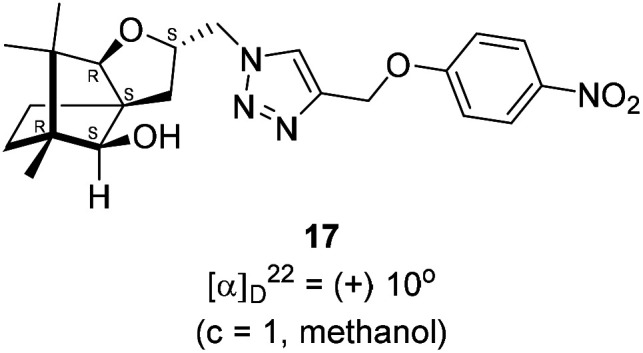
(2*S*,3a*R*,6*S*,7a*R*,8*S*)-(+)-2-(Azidomethyl)-6,7,7-trimethylhexahydro-2*H*-3a,6-methanobenzofuran-8-ol 16 (50.2 mg, 0.199 mmol) was dissolved in dichloromethane(DCM) (3 mL) and then 4-nitrophenylpropargyl ether (35.3 mg, 0.199 mmol), copper sulphate pentahydrate (0.67 mg, 0.004 mmol), sodium ascorbate (3.8 mg, 0.019 mmol) were added and then stirred at rt for 24 h. After the completion of reaction (by TLC, *R*_f_ = 0.46 (10% EtOAc/hexane)) the crude product was extracted using DCM (15 mL). The DCM layer was washed with water (2 × 15 mL), brine solution (2 × 15 mL) and then dried over anhydrous sodium sulfate. Then concentrated crude product was purified by column chromatography using silica (100–200 mesh) and increasing amount of 3 to 6% EtOAc in hexanes afforded (2*S*,3a*R*,6*S*,7a*R*,8*S*)-(+)-6,7,7-trimethyl-2-((4-((4-nitrophenoxy)methyl)-1*H*-1,2,3-triazol-1-yl)methyl)hexahydro-2*H*-3a,6-methanobenzofuran-8-ol 17 as a semisolid (71.6 mg, 84% yield); [*α*]^22^_D_ = +10° (*c* = 1, MeOH); IR (KBr, cm^−1^) 3416, 2953, 2872, 1597, 1509, 1339, 1259, 1173, 1112, 1049, 997, 853, 489. ^1^H NMR (400 MHz, CDCl_3_) *δ* 8.18 (d, *J* = 9.3 Hz, 2H), 7.76 (s, 1H), 7.08 (d, *J* = 9.3 Hz, 2H), 5.31 (s, 2H), 4.64 (dd, *J* = 7.3, 3.8 Hz, 1H), 4.48 (dd, *J* = 14.2, 3.7 Hz, 1H), 4.33 (dd, *J* = 14.1, 7.0 Hz, 1H), 3.33 (s, 1H), 3.21 (d, *J* = 1.7 Hz, 1H), 2.26 (dd, *J* = 13.1, 7.6 Hz, 1H), 1.68–1.61 (m, 2H), 1.25 (s, 1H), 1.18–1.13 (m, 1H), 1.06 (s, 3H), 0.99 (dd, *J* = 5.8, 2.3 Hz, 1H), 0.93 (s, 3H), 0.91 (s, 3H).·^13^C NMR (400 MHz, CDCl_3_ + CCl_4_) *δ* 163.2 (C), 143.0 (C), 142.2 (C), 126.1 (CH), 124.1 (CH), 115.1 (CH), 96.4 (CH), 88.6 (CH), 79.7 (CH), 62.6 (CH_2_), 56.6 (C), 54.3 (CH_2_), 50.9 (C), 43.4 (C), 31.4 (CH_2_), 29.7 (CH_2_), 26.7 (CH_3_), 23.8 (CH_2_), 21.5 (CH_3_), 13.4 (CH_3_); HRMS (ESI) calcd for C_22_H_29_N_4_O_5_ [M + H]^+^ 429.2138, found 429.2119.

## Conflicts of interest

There are no conflicts to declare.

## Supplementary Material

RA-011-D0RA09839F-s001

RA-011-D0RA09839F-s002
