# Supplementary material for: Studies in the rearrangement reactions involving camphorquinone
Source: RSC Adv. 2021 Feb 11;11(13):7180–6. doi: 10.1039/d0ra09839f (PMC8694982; doi:10.1039/d0ra09839f)
Supplement: RA-011-D0RA09839F-s001 [file RA-011-D0RA09839F-s001.pdf]

Electronic Supplementary Material (ESI) for RSC Advances.
This journal is © The Royal Society of Chemistry 2021

Supporting information

Title: Studies in the rearrangement reactions involving camphorquinone

H. Surya Prakash Rao,*[a,b] Ahana Saha[a], Satish Vijjapu[a].

[a] Department of Chemistry, Pondicherry University, Puducherry 605 014, India

[b] Syntho-Cascade Research Laboratories, Plot No: 65A &B, Survey No: 125, IDA Mallapur, Nacharam, Hyderabad-500076.

E-mail: profhspr@gmail.com, hsprlab@gmail.com; Tel: +91-9443264222

Table of contents:

^{1}H NMR (400 MHz, $CDCl_3$ + CCl_4; 1:1), ^{13}C NMR (100 MHz, $CDCl_3$ + CCl_4; 1:1), DEPT–135 (100 MHz, $CDCl_3$ + CCl_4; 1:1) and HRMS spectra of (1*R*,3*R*,4*S*)-(+)-3-allyl-3-hydroxy-1,7,7-trimethylbicyclo[2.2.1]heptan-2-one **5**. S3-S4

^{1}H NMR (400 MHz, $CDCl_3$ + CCl_4; 1:1), ^{13}C NMR (100 MHz, $CDCl_3$ + CCl_4; 1:1), DEPT–135 (100 MHz, $CDCl_3$ + CCl_4; 1:1) and HRMS spectra of (1*R*,2*S*,3*R*,4*S*)-(-)-3-allyl-1,7,7-trimethylbicyclo[2.2.1]heptane-2,3-diol **6**. S5-S6

^{1}H NMR (400 MHz, $CDCl_3$ + CCl_4; 1:1), ^{13}C NMR (100 MHz, $CDCl_3$ + CCl_4; 1:1), DEPT–135 (100 MHz, $CDCl_3$ + CCl_4; 1:1) and HRMS spectra of (3a*R*,4*S*,7*R*,7a*S*)-(-)-3a-allyl-2,2,7,8,8-pentamethylhexahydro-4,7-methanobenzo[d]-[1,3]dioxole **7**. S7-S8

^{1}H NMR (400 MHz, $CDCl_3$ + CCl_4; 1:1), ^{13}C NMR (100 MHz, $CDCl_3$ + CCl_4; 1:1), DEPT–135 (100 MHz, $CDCl_3$ + CCl_4; 1:1), 2D NMR (HSQC, HMBC and COSY), HRMS spectra and ORTEP diagram of (2*S*,3a*R*,6*S*,7a*R*,8*S*)-(-)-2-(iodomethyl)-6,7,7-trimethylhexahydro-2*H*-3a,6-methanobenzofuran-8-ol **4**. S9-S12

^{1}H NMR (400 MHz, $CDCl_3$ + CCl_4; 1:1), ^{13}C NMR (100 MHz, $CDCl_3$), DEPT–135 (100 MHz, $CDCl_3$ + CCl_4; 1:1) and HRMS spectra of (2*S*,3a*R*,6*S*,7a*R*,8*S*)-(-)2-(bromomethyl)-6,7,7-trimethylhexahydro-2*H*-3a,6-methanobenzofuran-8-ol **8**. S13-S14

^{1}H NMR (400 MHz, $CDCl_3$ + CCl_4; 1:1), ^{13}C NMR (100 MHz, $CDCl_3$ + CCl_4; 1:1), DEPT–135 (100 MHz, $CDCl_3$ + CCl_4; 1:1) and HRMS spectra of (1*R*,3*R*,4*S*)-(+)-3-hydroxy-1,7,7-trimethyl-3-(propa-1,2-dien-lyl)bicyclo[2,2,1]heptan-2-one **14**. S15-S16

^{1}H NMR (400 MHz, $CDCl_3$ + CCl_4; 1:1), ^{13}C NMR (100 MHz, $CDCl_3$ + CCl_4; 1:1), DEPT–135 (100 MHz, $CDCl_3$ + CCl_4; 1:1) and HRMS spectra and ORTEP diagram of (1*R*,2*S*,3*R*,4*S*)-(+)-1,7,7-trimethyl-3-(propa-1,2-dien-lyl)bicyclo[2.2.1]heptane-2,3-diol **15**. S17-S19

^{1}H NMR (400 MHz, $CDCl_3$ + CCl_4; 1:1), ^{13}C NMR (100 MHz, $CDCl_3$ + CCl_4; 1:1), DEPT–135 (100 MHz, $CDCl_3$ + CCl_4; 1:1), 2D NMR (HSQC, HMBC and COSY) and HRMS spectra of (2*S*,3a*R*,6*S*,7a*R*,8*S*)-(+)-2-(azidomethyl)-6,7,7-trimethylhexahydro-2*H*-3a,6-methanobenzofuran-8-ol **16**. S19-S22

^{1}H NMR (400 MHz, $CDCl_3$ + CCl_4; 1:1), ^{13}C NMR (100 MHz, $CDCl_3$ + CCl_4; 1:1), DEPT–135 (100 MHz, $CDCl_3$ + CCl_4; 1:1) and HRMS spectra of (2*S*,3a*R*,6*S*,7a*R*,8*S*)-(+)-6,7,7-trimethyl-2-((4-((4nitrophenoxy)methyl)-1*H*-1,2,3-triazol-1yl)methyl)hexahydro-2*H*-3a,6-methanobenzofuran-8-ol **17**. S23-S24

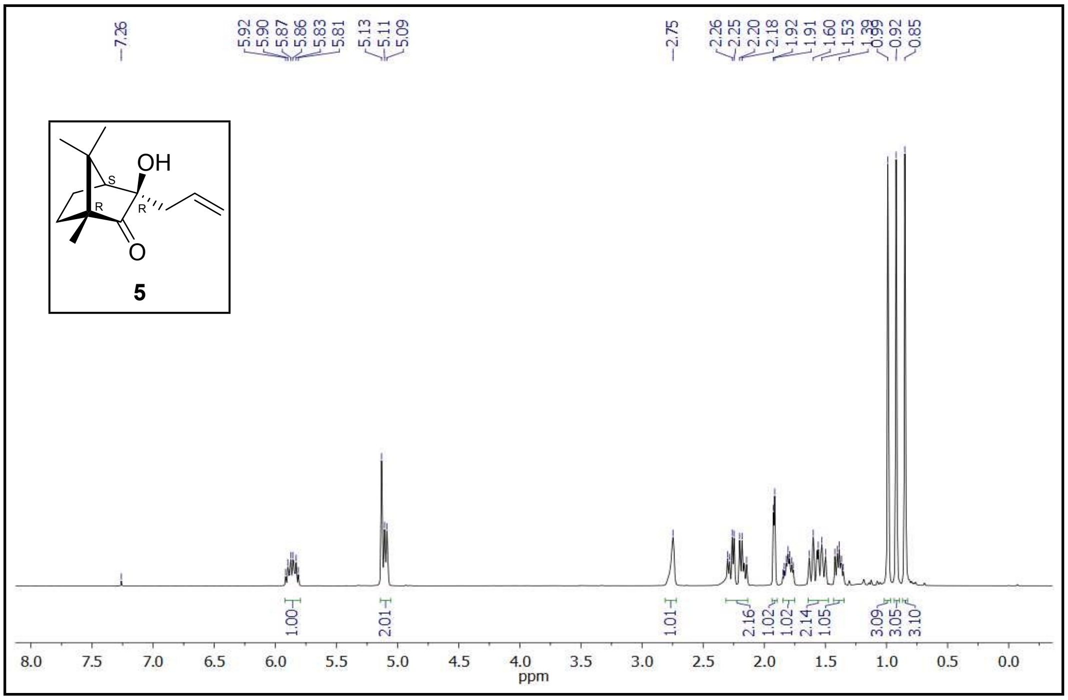

^{1}H NMR (400 MHz, $CDCl_3$ + CCl_4; 1:1) spectrum of (1*R*,3*R*,4*S*)-3-allyl-3-hydroxy-1,7,7-trimethylbicyclo[2.2.1]heptan-2-one **5**.

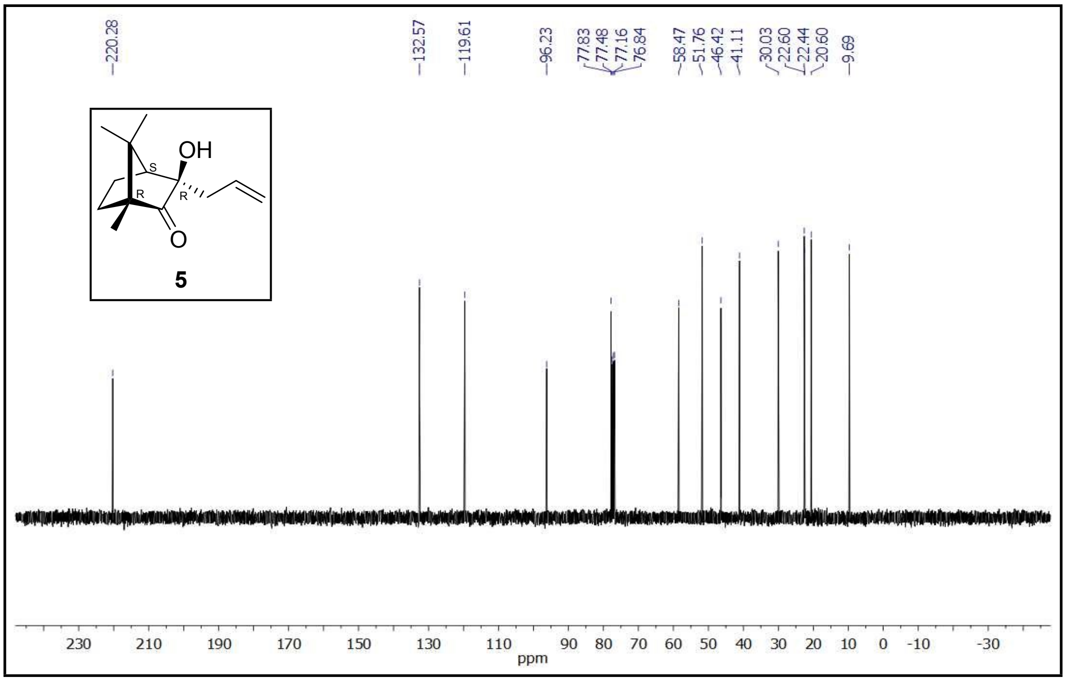

^{13}C NMR (100 MHz, $CDCl_3$ + CCl_4; 1:1) spectrum of (1*R*,3*R*,4*S*)-3-allyl-3-hydroxy-1,7,7-trimethylbicyclo[2.2.1]heptan-2-one **5**.

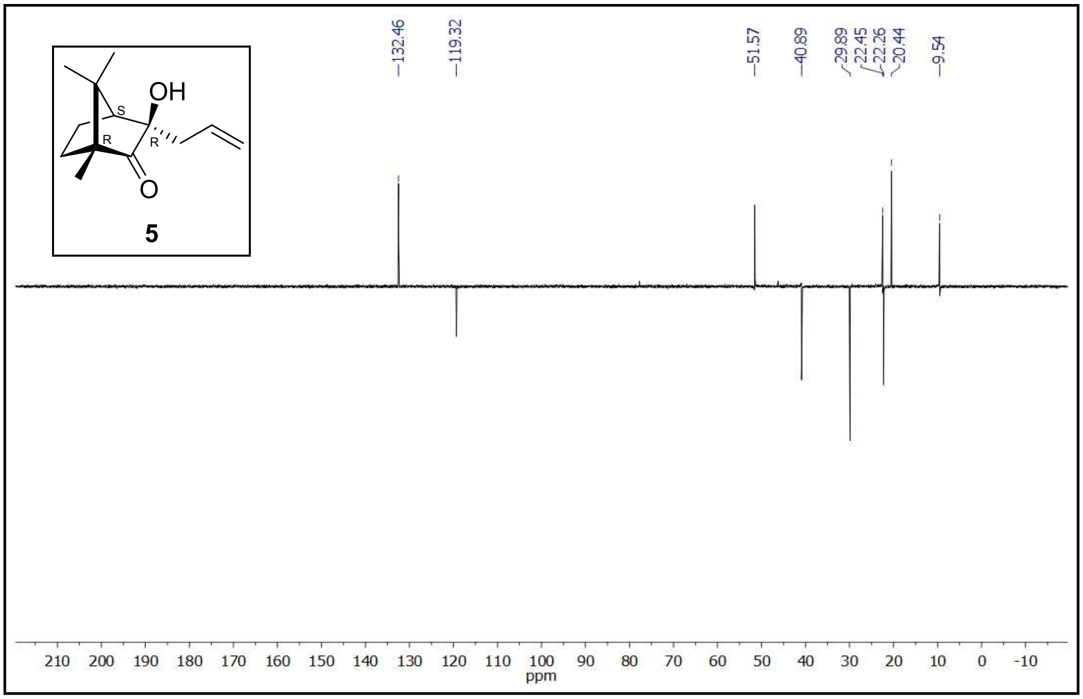

DEPT-135 NMR (100 MHz, $CDCl_3$ + CCl_4; 1:1) spectrum of (1*R*,3*R*,4*S*)-3-allyl-3-hydroxy-1,7,7-trimethylbicyclo[2.2.1]heptan-2-one **5**.

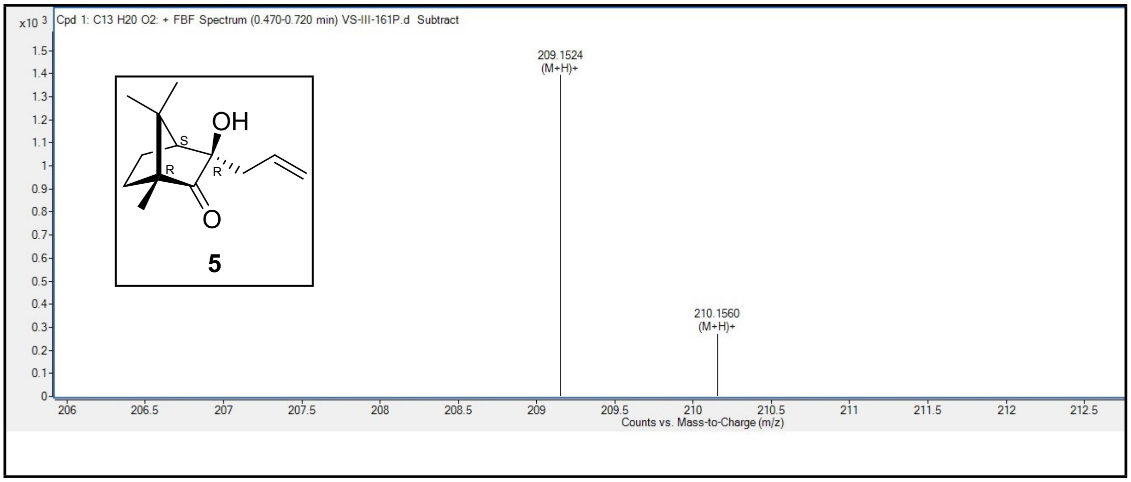

HRMS spectrum of (1*R*,3*R*,4*S*)-3-allyl-3-hydroxy-1,7,7-trimethylbicyclo[2.2.1]heptan-2-one **5**.

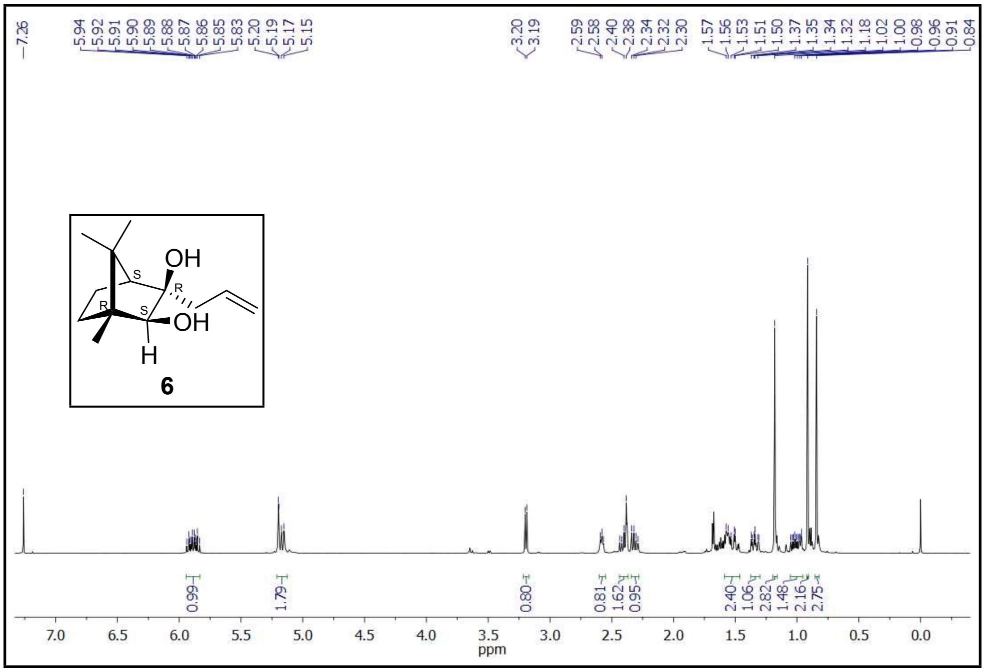

^{1}H NMR (400 MHz, $CDCl_3$ + CCl_4; 1:1) spectrum of (1*R*,2*S*,3*R*,4*S*)-(-)-3-allyl-1,7,7-trimethylbicyclo[2.2.1]heptane-2,3-diol **6**.

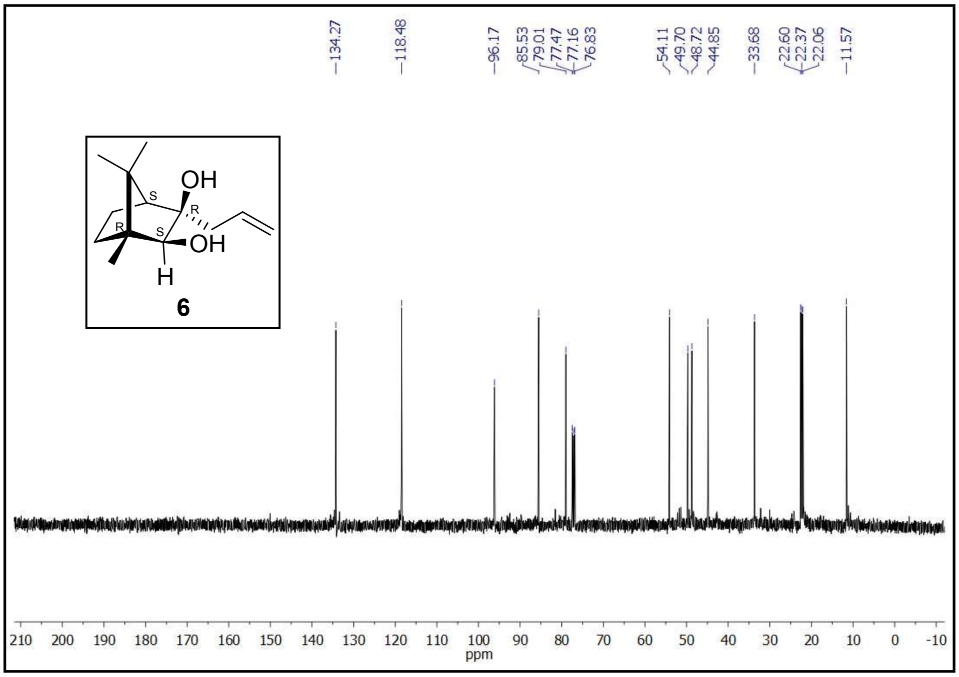

^{13}C NMR (100 MHz, $CDCl_3$ + CCl_4; 1:1) spectrum of (1*R*,2*S*,3*R*,4*S*)-(-)-3-allyl-1,7,7-trimethylbicyclo[2.2.1]heptane-2,3-diol **6**.

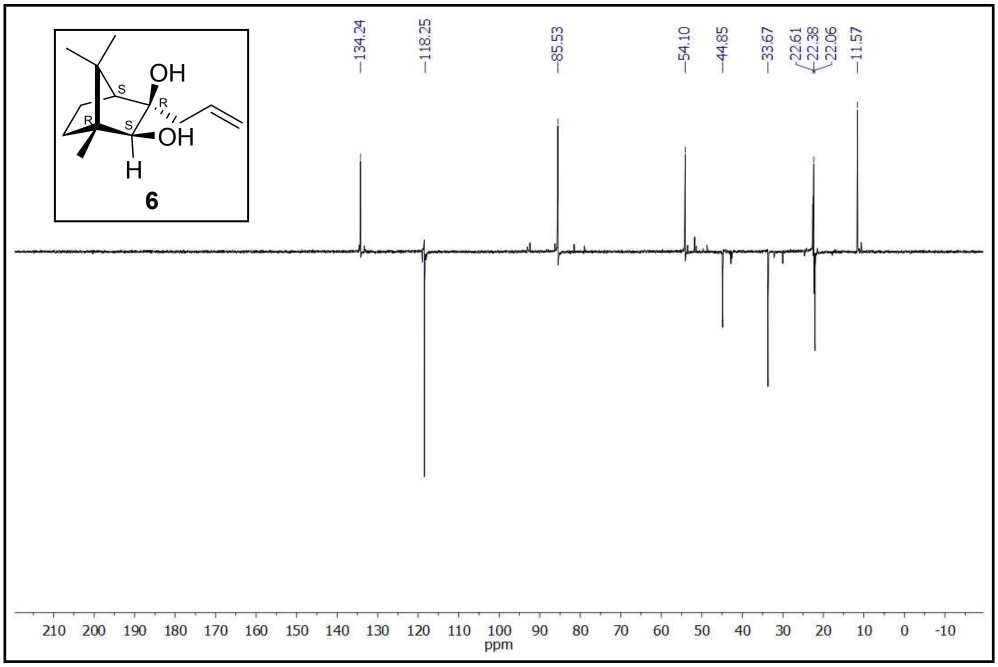

DEPT-135 NMR (100 MHz, $CDCl_3$ + CCl_4; 1:1) spectrum of (1*R*,2*S*,3*R*,4*S*)-(-)-3-allyl-1,7,7-trimethylbicyclo[2.2.1]heptane-2,3-diol **6**.

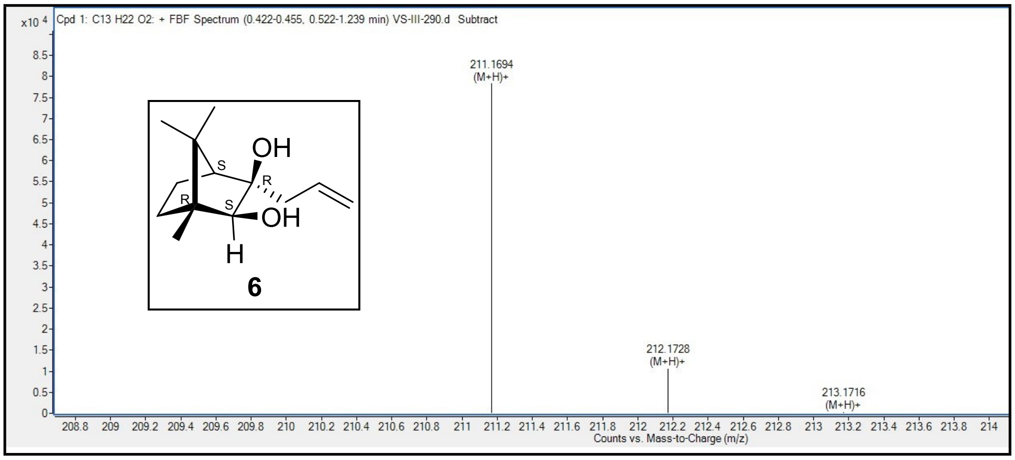

HRMS spectrum of (1*R*,2*S*,3*R*,4*S*)-(-)-3-allyl-1,7,7-trimethylbicyclo[2.2.1]heptane-2,3-diol **6**.

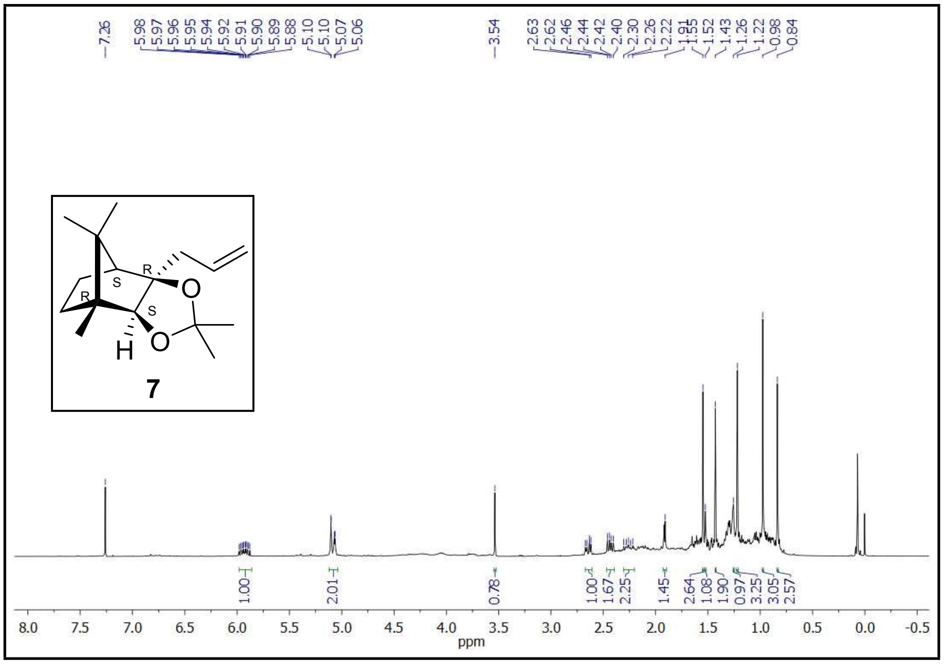

^{1}H NMR (400 MHz, $CDCl_3$ + CCl_4; 1:1) spectrum of (3a*R*,4*S*,7*R*,7a*S*)-3a-allyl-2,2,7,8,8-pentamethylhexahydro-4,7-methanobenzo[d]-[1,3]dioxole **7**.

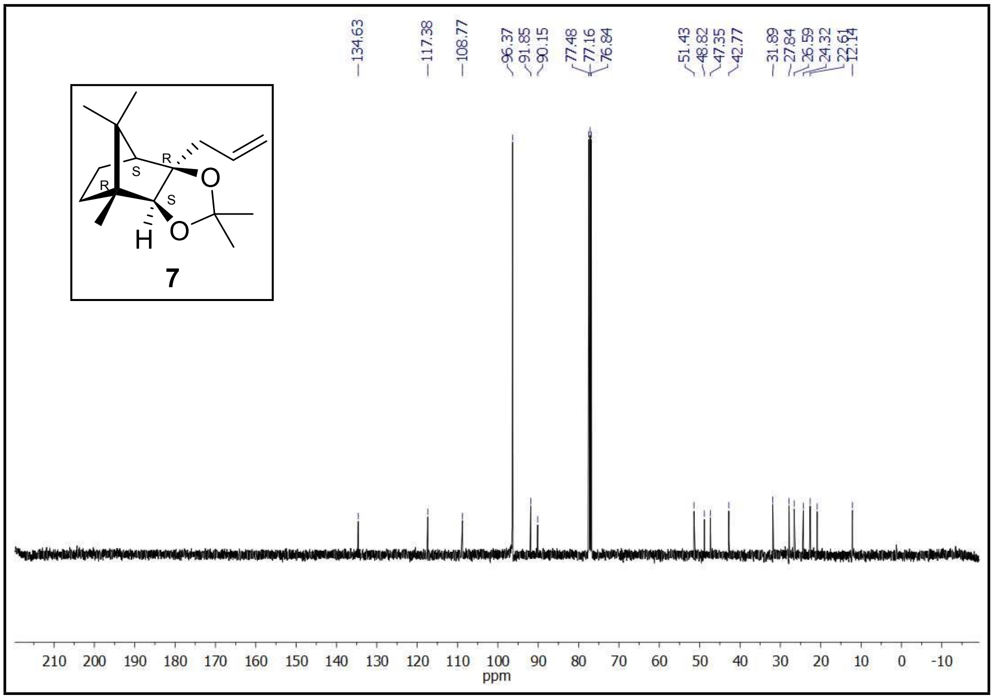

^{13}C NMR (100 MHz, $CDCl_3$ + CCl_4; 1:1) spectrum of (3a*R*,4*S*,7*R*,7a*S*)-3a-allyl-2,2,7,8,8-pentamethylhexahydro-4,7-methanobenzo[d]-[1,3]dioxole **7**.

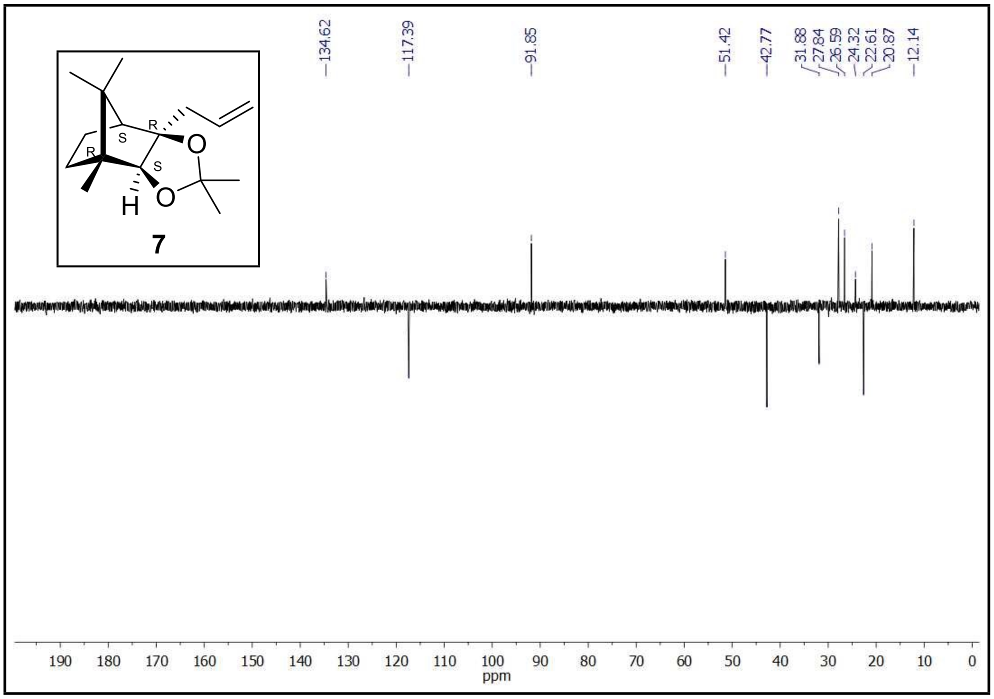

DEPT-135 NMR (100 MHz, $CDCl_3$ + CCl_4; 1:1) spectrum of (3a*R*,4*S*,7*R*,7a*S*)-3a-allyl-2,2,7,8,8-pentamethylhexahydro-4,7-methanobenzo[d]-[1,3]dioxole **7**.

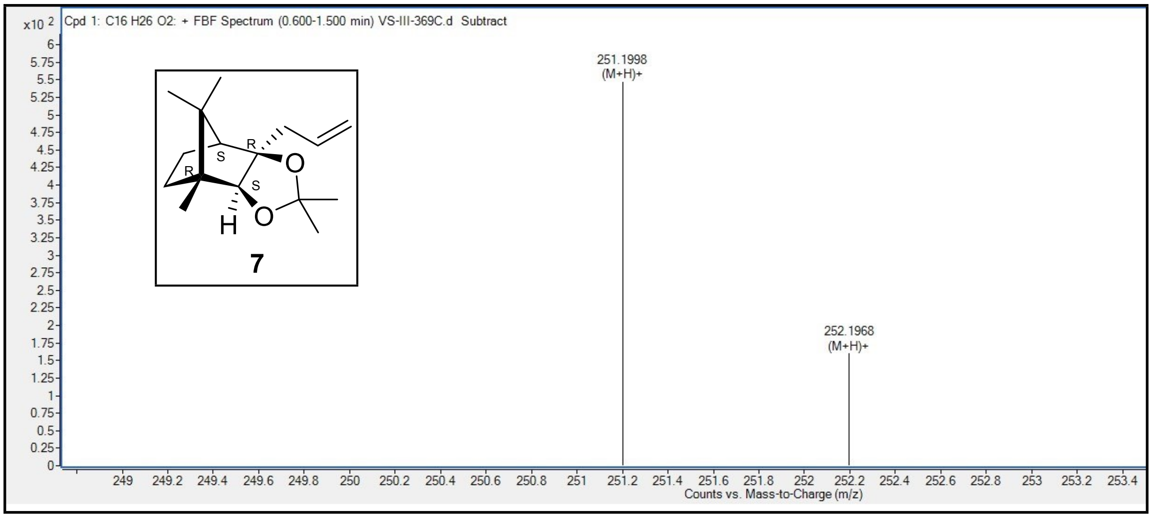

HRMS spectrum of (3a*R*,4*S*,7*R*,7a*S*)-3a-allyl-2,2,7,8,8-pentamethylhexahydro-4,7-methanobenzo[d]-[1,3]dioxole **7**.

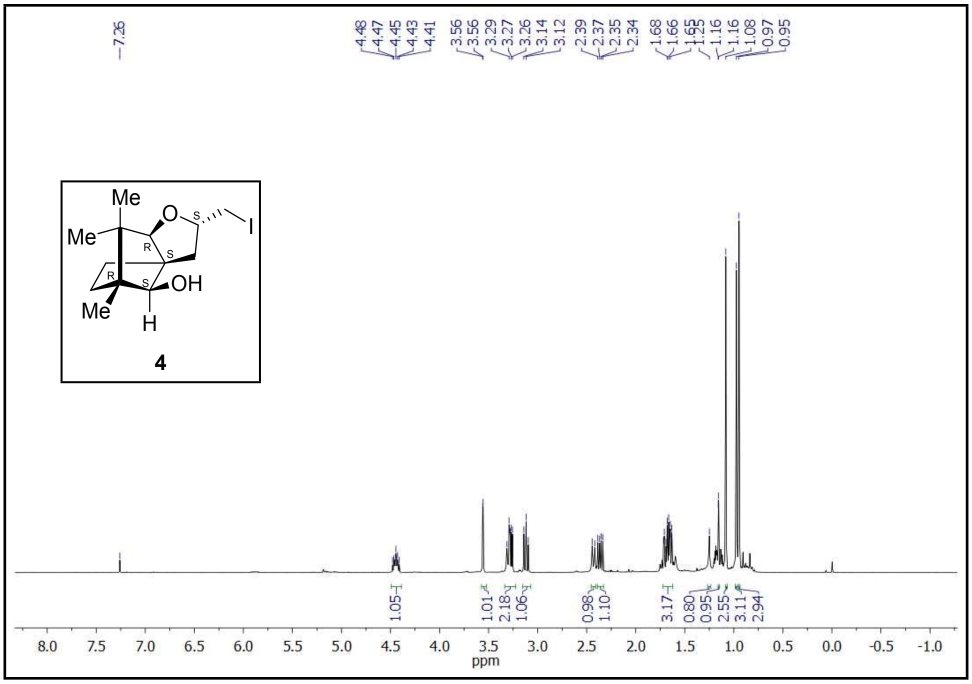

^{1}H NMR (400 MHz, $CDCl_3$ + CCl_4; 1:1) spectrum of (2*S*,3a*R*,6*S*,7a*R*,8*S*)-2-(iodomethyl)-6,7,7-trimethylhexahydro-2*H*-3a,6-methanobenzofuran-8-ol **4**.

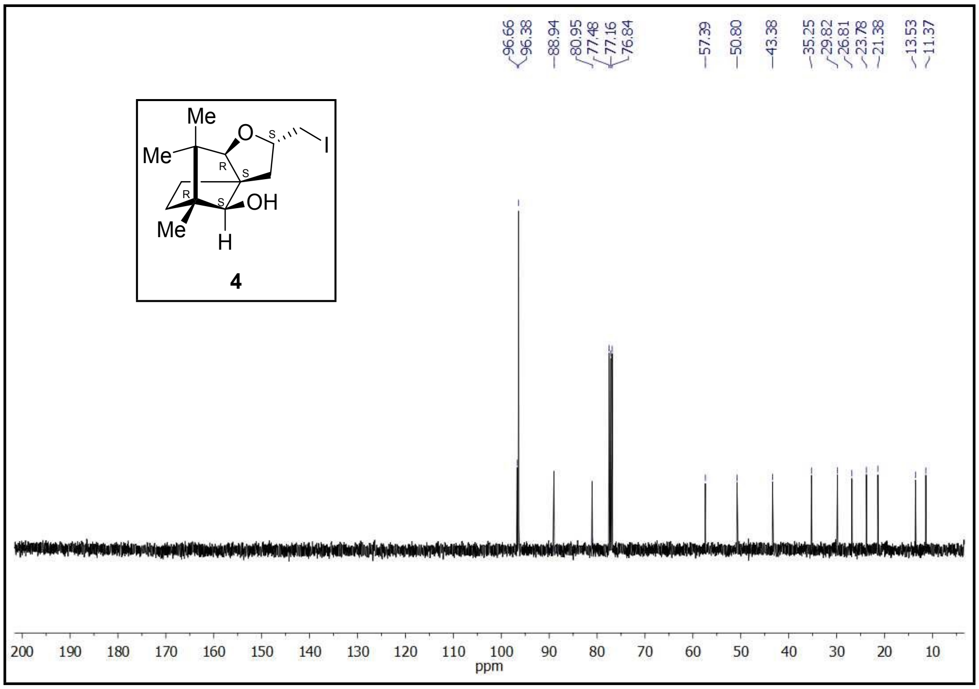

^{13}C NMR (100 MHz, $CDCl_3$ + CCl_4; 1:1) spectrum of (2*S*,3a*R*,6*S*,7a*R*,8*S*)-2-(iodomethyl)-6,7,7-trimethylhexahydro-2*H*-3a,6-methanobenzofuran-8-ol **4**.

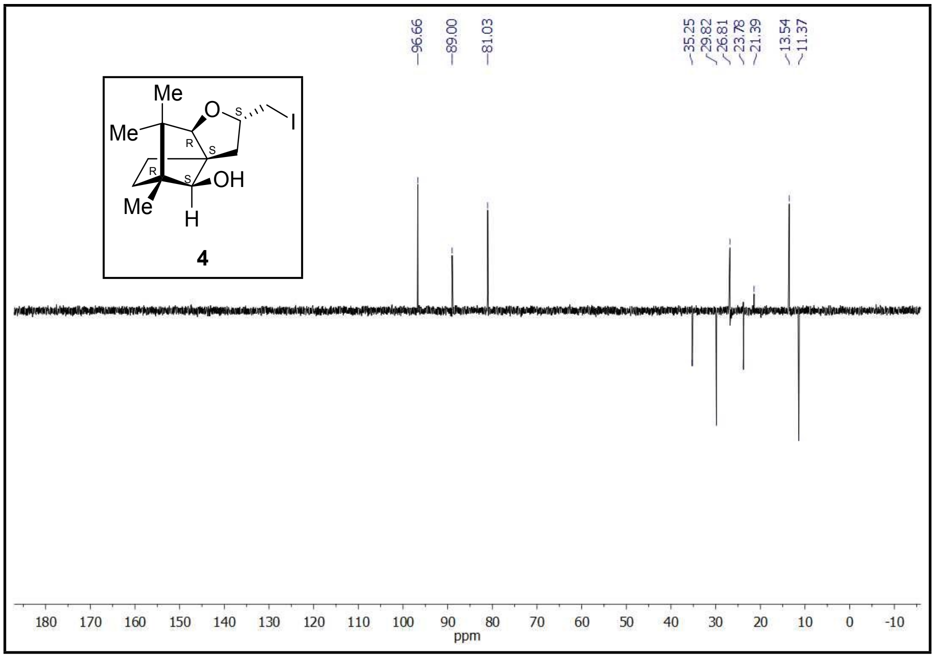

DEPT-135 NMR (100 MHz, $CDCl_3$ + CCl_4; 1:1) spectrum of (2*S*,3a*R*,6*S*,7a*R*,8*S*)-2-(iodomethyl)-6,7,7-trimethylhexahydro-2*H*-3a,6-methanobenzofuran-8-ol **4**.

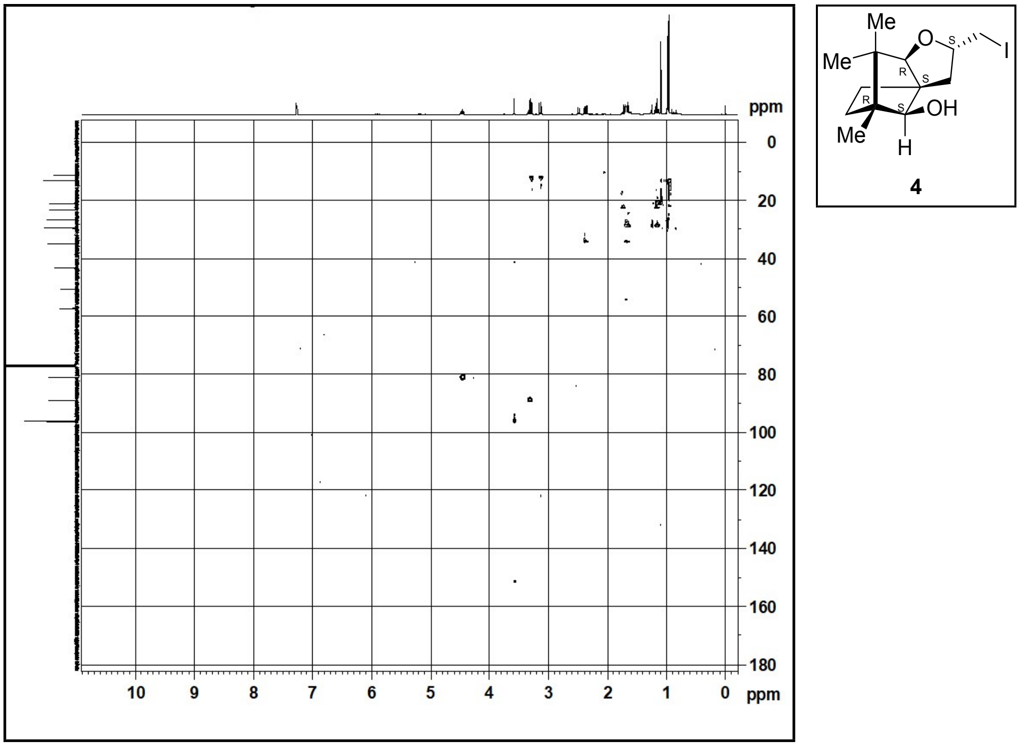

HSQC spectrum of (2*S*,3a*R*,6*S*,7a*R*,8*S*)-2-(iodomethyl)-6,7,7-trimethylhexahydro-2*H*-3a,6-methanobenzofuran-8-ol **4**.

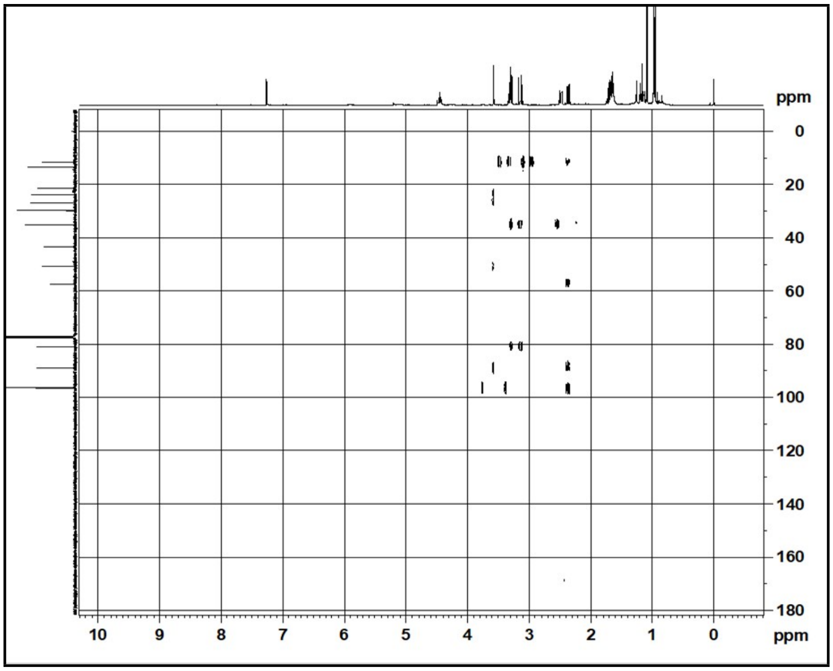

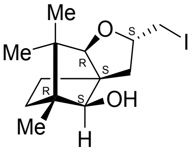

4

HMBC spectrum of (2*S*,3a*R*,6*S*,7a*R*,8*S*)-2-(iodomethyl)-6,7,7-trimethylhexahydro-2*H*-3a,6-methanobenzofuran-8-ol **4**.

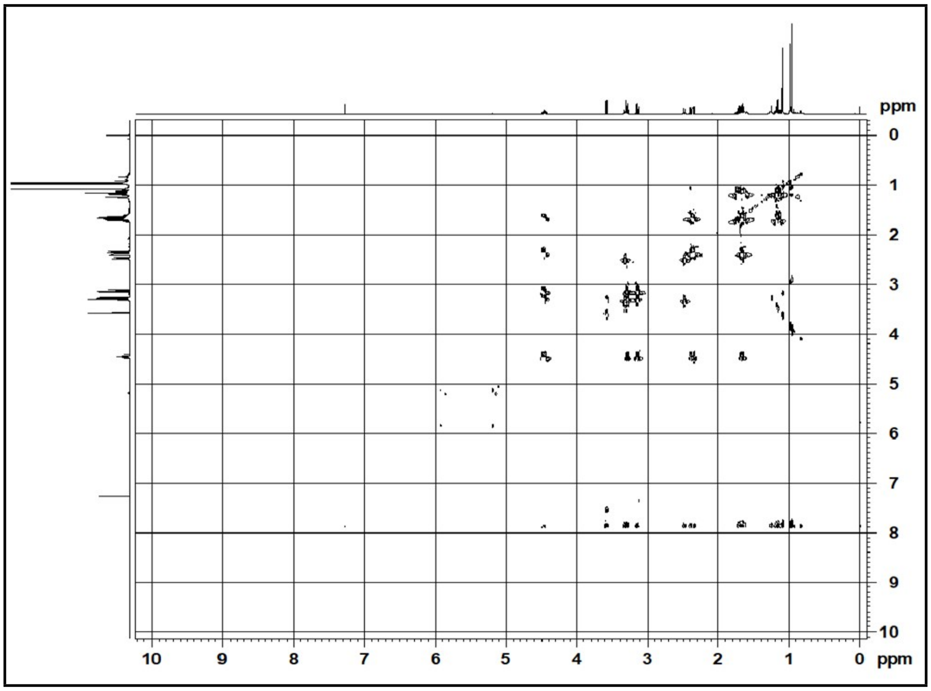

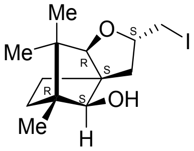

4

COSY spectrum of (2*S*,3a*R*,6*S*,7a*R*,8*S*)-2-(iodomethyl)-6,7,7-trimethylhexahydro-2*H*-3a,6-methanobenzofuran-8-ol **4**.

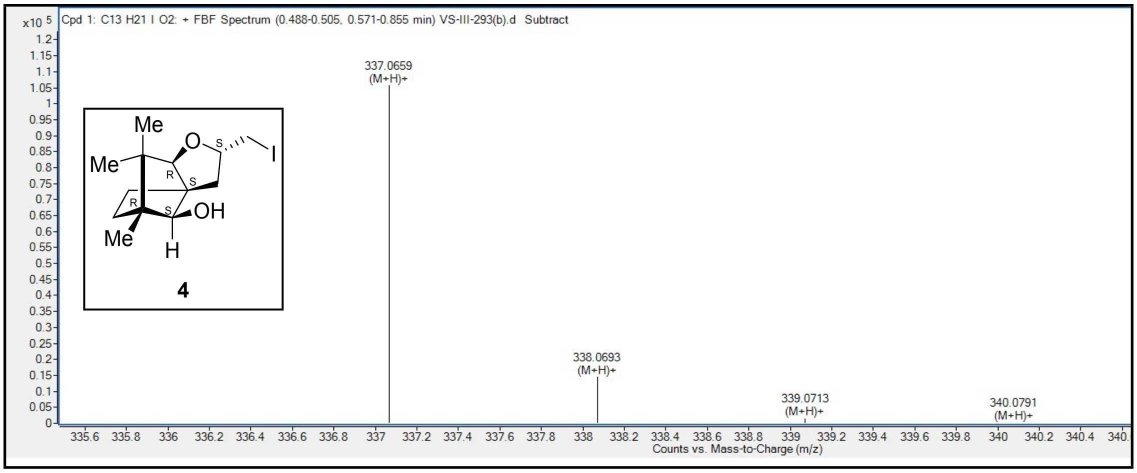

HRMS spectrum of (2*S*,3a*R*,6*S*,7a*R*,8*S*)-2-(iodomethyl)-6,7,7-trimethylhexahydro-2*H*-3a,6-methanobenzofuran-8-ol **4**.

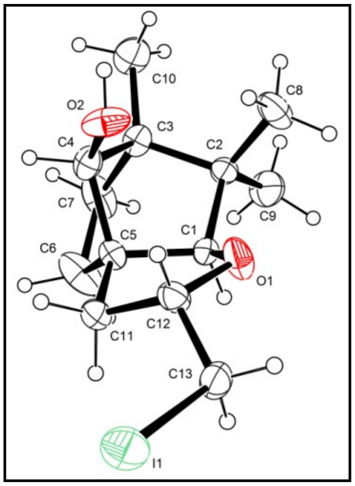

ORTEP diagram of (2*S*,3a*R*,6*S*,7a*R*,8*S*)-2-(iodomethyl)-6,7,7-trimethylhexahydro-2*H*-3a,6-methanobenzofuran-8-ol **4**.

Crystal data: Empirical formula, $C_{13}H_{21}IO_2$; Formula weight, 339.70; Crystal color, habit: colorless, rectangular block; Crystal system, triclinic; Crystal dimensions, 0.35 x 0.22 x 0.16 mm^3; Lattice parameters, a = 11.3843(5) Å, b = 11.4267(5) Å, c = 11.6597(5) Å; α = 99.91(19), β = 106.38(16), γ = 95.26(18); V = 1417.63(11) A^3; Space group P-1; Z = 4; D_{calcd} = 1.592 g/cm^3; F_{000} = 679; λ (M_o K_α) = 0.7107 Å; R ($I \geq 2\sigma_1$) = 0.0427, wR^2 = 0.1213. Detailed X-ray crystallographic data is available from the Cambridge Crystallographic Data Centre, 12 Union Road, Cambridge CB2 1EZ, UK (CCDC # 1402567).

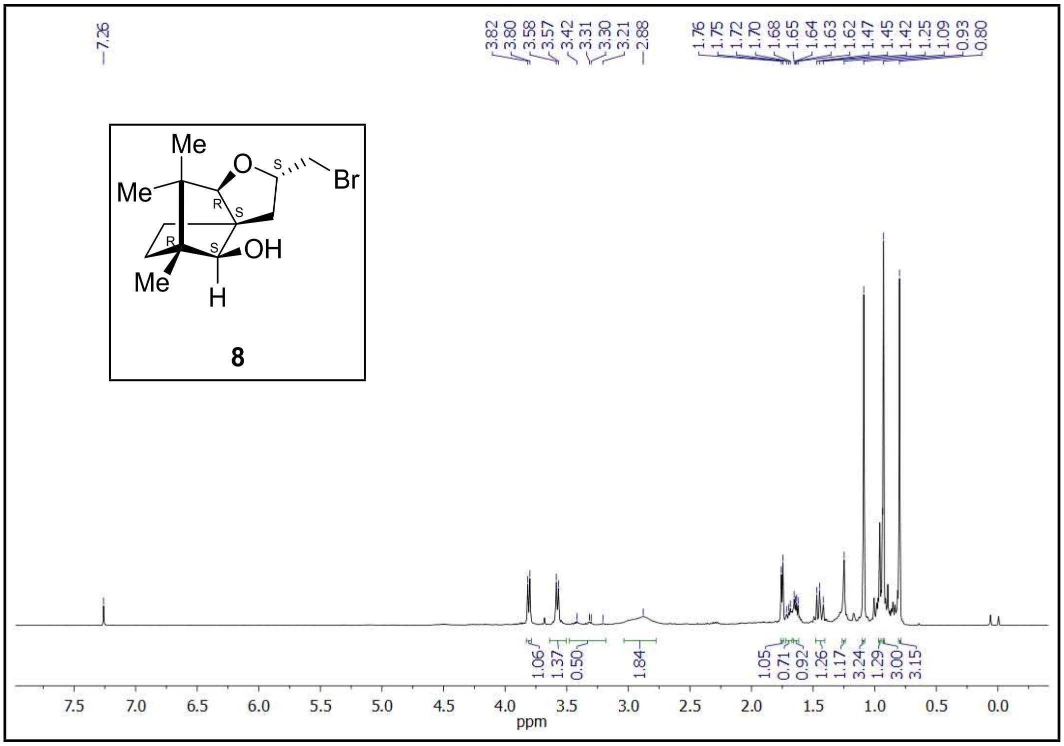

^{1}H NMR (400 MHz, $CDCl_3$ + CCl_4; 1:1) spectrum of (2*S*,3a*R*,6*S*,7a*R*,8*S*)-2-(bromomethyl)-6,7,7-trimethylhexahydro-2*H*-3a,6-methanobenzofuran-8-ol **8**.

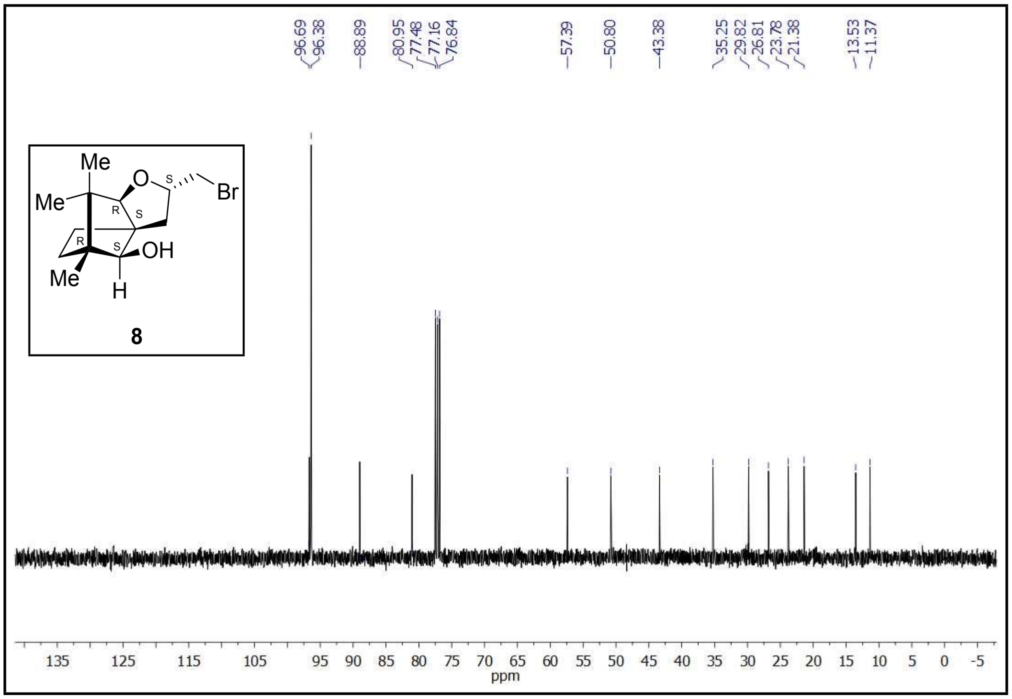

^{13}C NMR (100 MHz, $CDCl_3$ + CCl_4; 1:1) spectrum of (2*S*,3a*R*,6*S*,7a*R*,8*S*)-2-(bromomethyl)-6,7,7-trimethylhexahydro-2*H*-3a,6-methanobenzofuran-8-ol **8**.

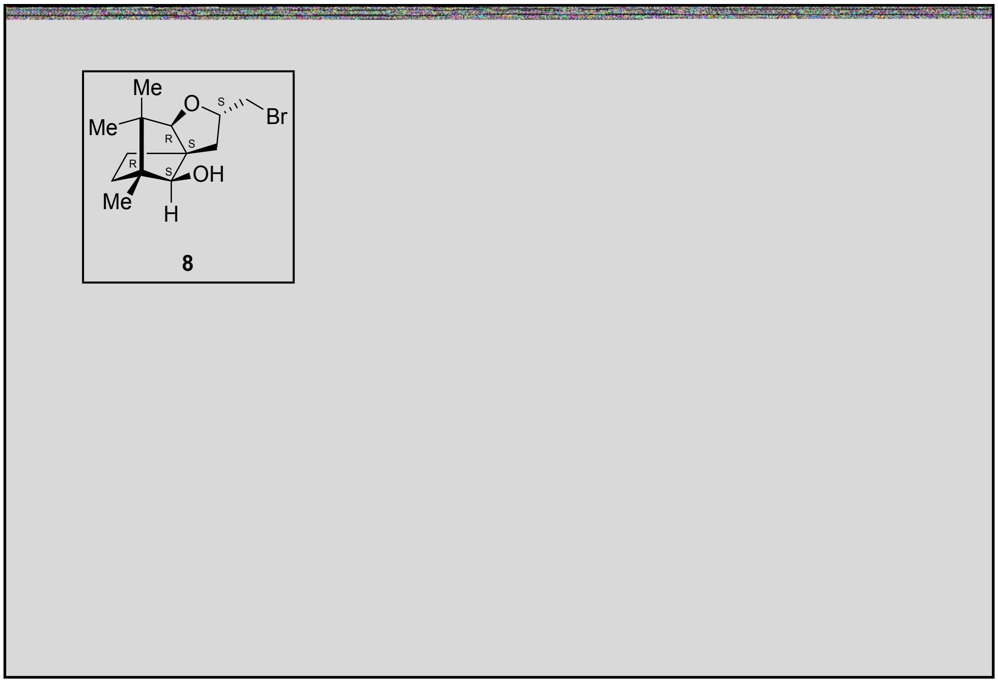

DEPT-135 NMR (100 MHz, $CDCl_3$ + CCl_4; 1:1) spectrum of (2*S*,3a*R*,6*S*,7a*R*,8*S*)-2-(bromomethyl)-6,7,7-trimethylhexahydro-2*H*-3a,6-methanobenzofuran-8-ol **8**.

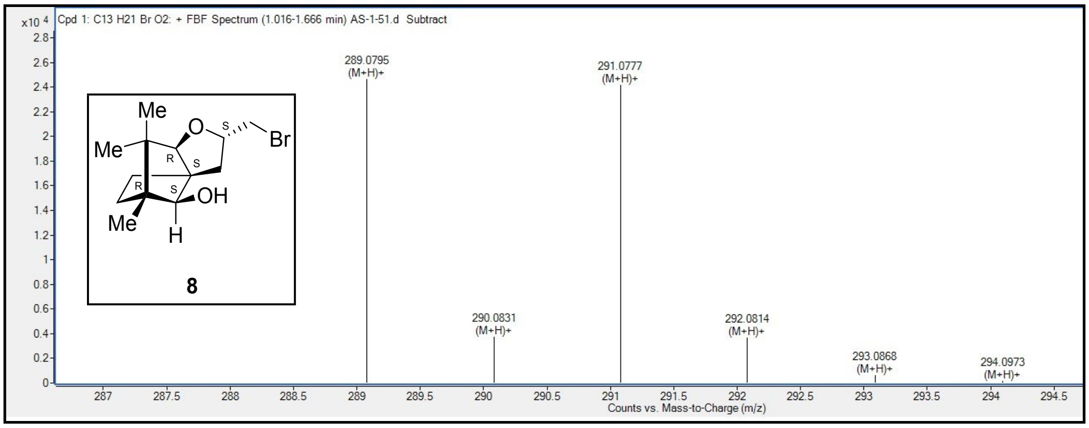

HRMS spectrum of (2*S*,3a*R*,6*S*,7a*R*,8*S*)-2-(bromomethyl)-6,7,7-trimethylhexahydro-2*H*-3a,6-methanobenzofuran-8-ol **8**.

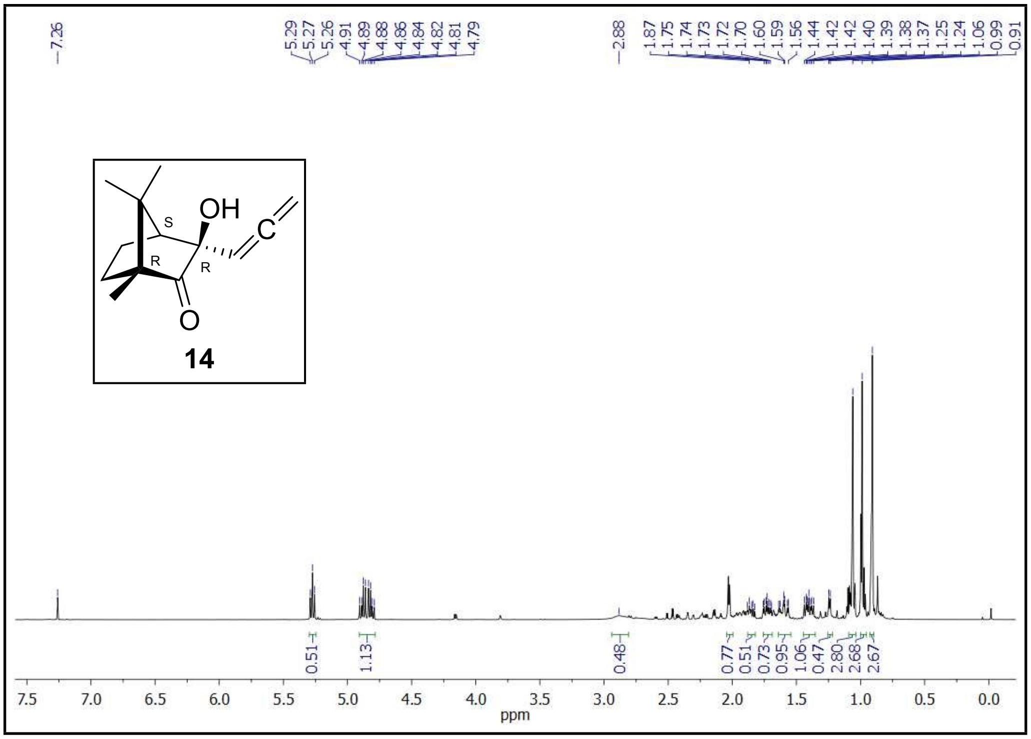

^1H NMR (400 MHz, $CDCl_3$ + CCl_4; 1:1) spectrum of (1*R*,3*R*,4*S*)-(+)-3-hydroxy-1,7,7-trimethyl-3-(propa-1,2-dien-lyl)bicyclo[2.2.1]-heptan-2-one **14**.

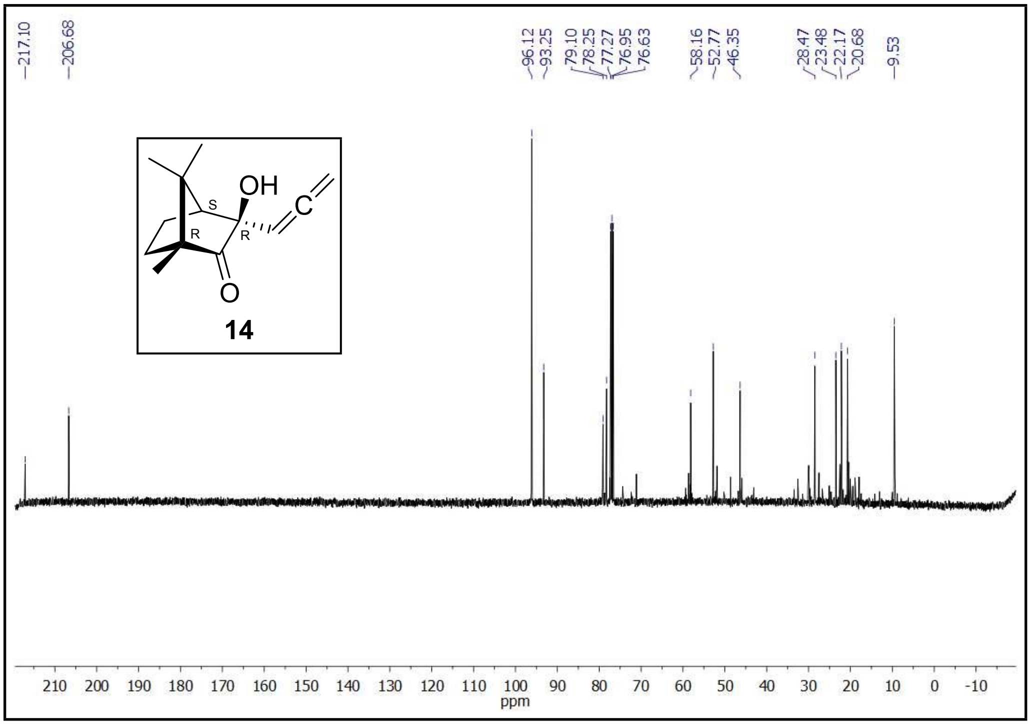

^{13}C NMR (100 MHz, $CDCl_3$ + CCl_4; 1:1) spectrum of (1*R*,3*R*,4*S*)-(+)-3-hydroxy-1,7,7-trimethyl-3-(propa-1,2-dien-lyl)bicyclo[2.2.1]-heptan-2-one **14**.

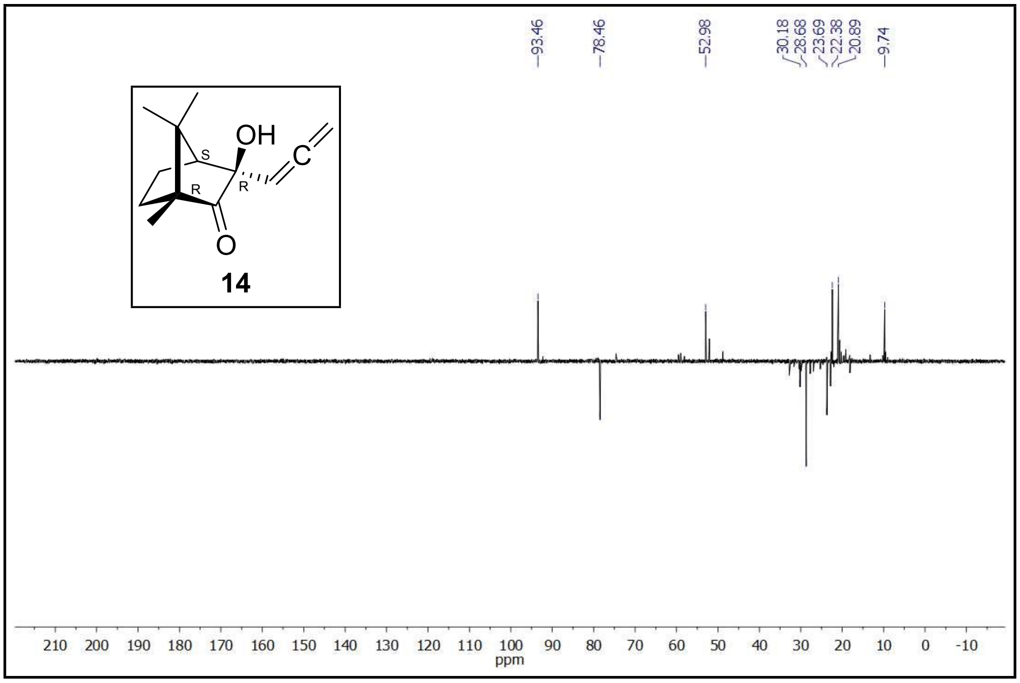

DEPT-135 NMR (100 MHz, $CDCl_3$ + CCl_4; 1:1) spectrum of (1*R*,3*R*,4*S*)-(+)-3-hydroxy-1,7,7-trimethyl-3-(propa-1,2-dien-lyl)bicyclo[2.2.1]-heptan-2-one **14**.

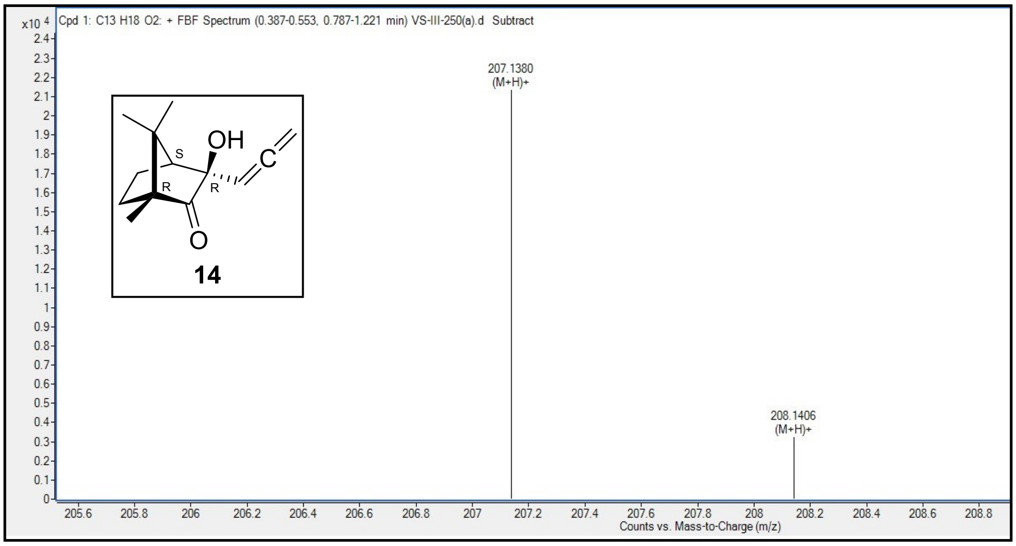

HRMS spectrum of (1*R*,3*R*,4*S*)-(+)-3-hydroxy-1,7,7-trimethyl-3-(propa-1,2-dien-lyl)bicyclo[2.2.1]-heptan-2-one **14**.

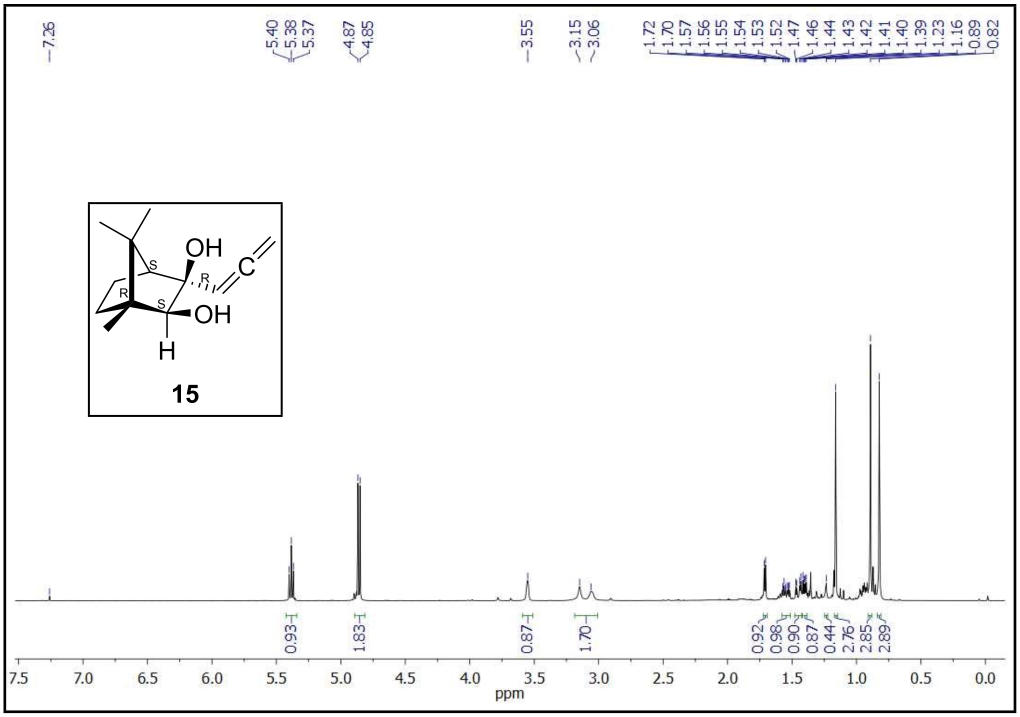

^{1}H NMR (400 MHz, $CDCl_3$ + CCl_4; 1:1) spectrum of (1*R*,2*S*,3*R*,4*S*)-(+)-1,7,7-trimethyl-3-(propa-1,2-dien-lyl)bicyclo[2.2.1]heptane-2,3-diol **15**.

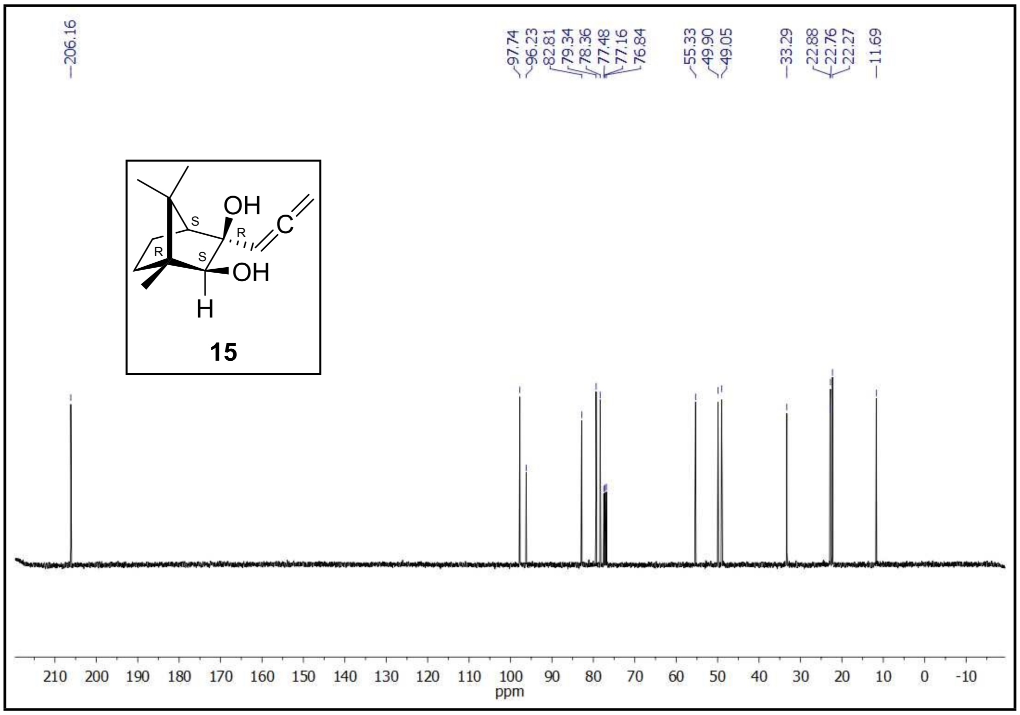

^{13}C NMR (100 MHz, $CDCl_3$ + CCl_4; 1:1) spectrum of (1*R*,2*S*,3*R*,4*S*)-(+)-1,7,7-trimethyl-3-(propa-1,2-dien-lyl)bicyclo[2.2.1]heptane-2,3-diol **15**.

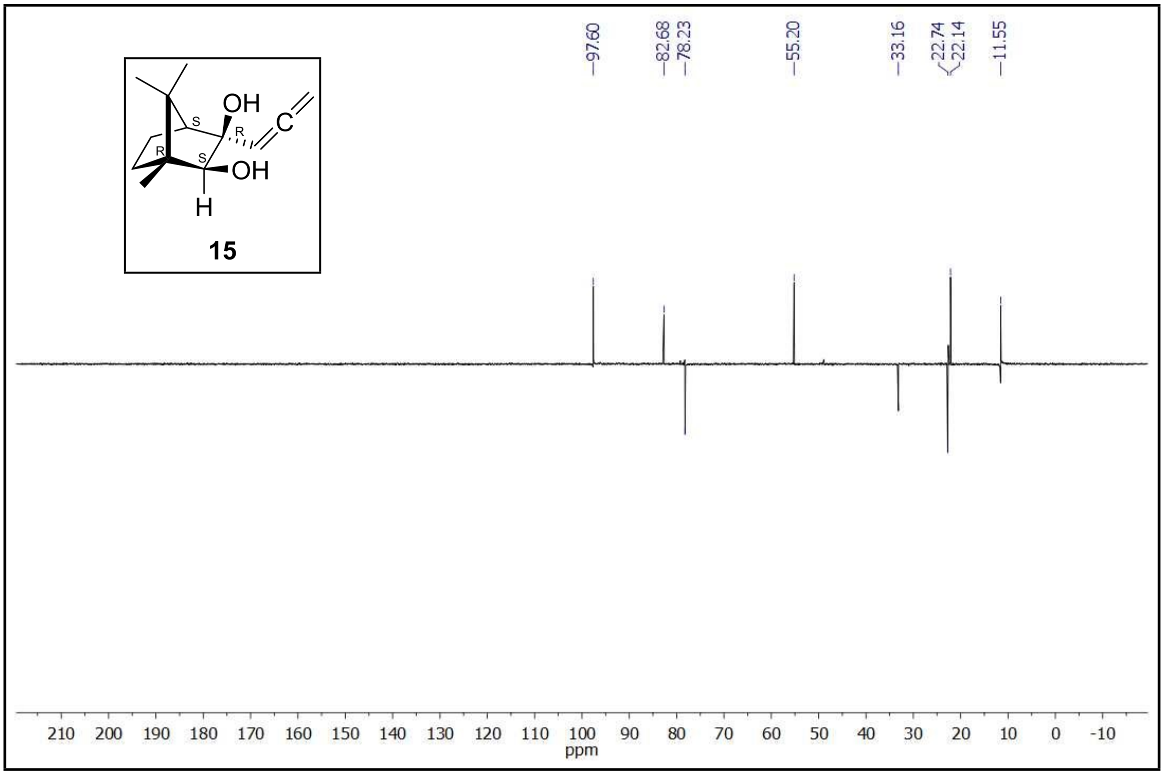

DEPT-135 NMR (100 MHz, $CDCl_3$ + CCl_4; 1:1) spectrum of (1*R*,2*S*,3*R*,4*S*)-(+)-1,7,7-trimethyl-3-(propa-1,2-dien-lyl)bicyclo[2.2.1]heptane-2,3-diol **15**.

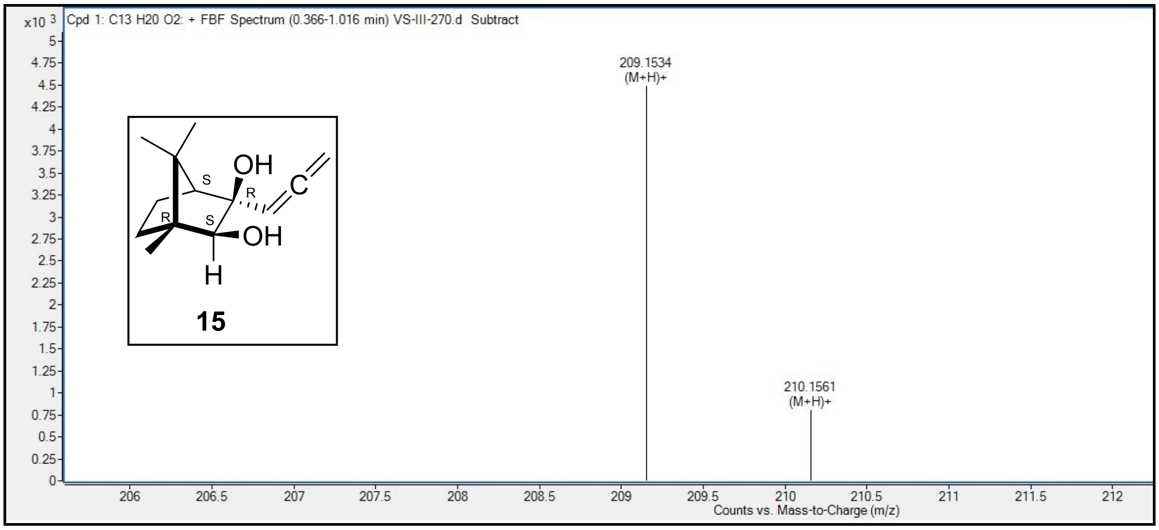

HRMS spectrum of (1*R*,2*S*,3*R*,4*S*)-(+)-1,7,7-trimethyl-3-(propa-1,2-dien-lyl)bicyclo[2.2.1]heptane-2,3-diol **15**.

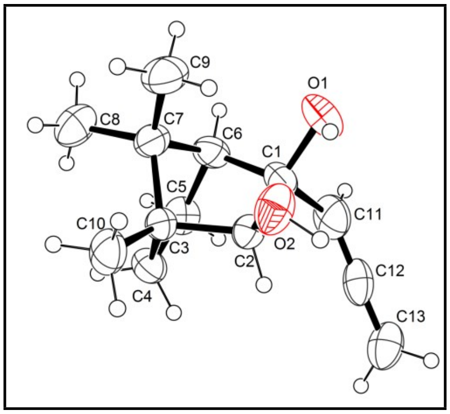

ORTEP diagram of (1*R*,2*S*,3*R*,4*S*)-(+)-1,7,7-trimethyl-3-(propa-1,2-dien-1-yl)bicyclo[2.2.1]heptane-2,3-diol **15**.

Crystal data: Empirical formula, $C_{13}H_{20}O_2$; Formula weight, 208.29; Crystal color, habit: colorless, plate; Crystal system, orthorhombic; Crystal dimensions, 0.4 x 0.3 x 0.04 mm^3; Lattice parameters, a = 6.9420(8) Å, b = 10.4314(12) Å, c = 16.881(2) Å; α = 90.00, β = 90.00, γ = 90.00; V = 1222.4(2) A^3; Space group P2-1; Z = 5; D_{calcd} = 1.415 g/cm^3; F_{000} = 570; λ(Mo Kα) = 0.7107 Å; R ($I \geq 2\sigma_1$) = 0.0542, wR^2 = 0.1209. Detailed X-ray crystallographic data is available from the Cambridge Crystallographic Data Centre, 12 Union Road, Cambridge CB2 1EZ, UK (CCDC # 1402568).

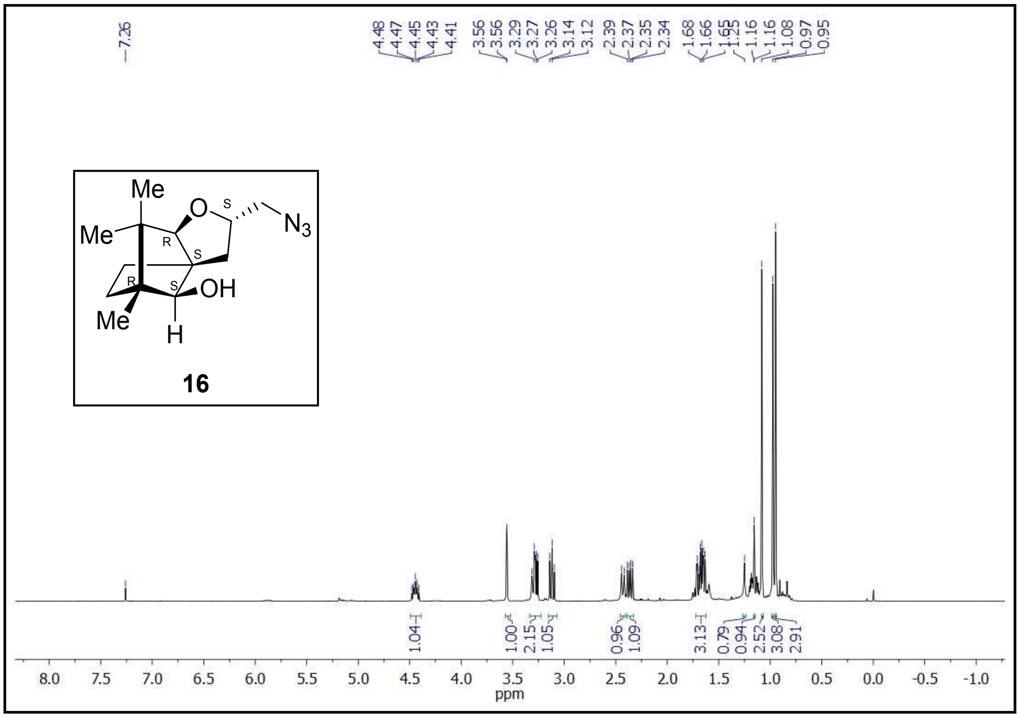

^{1}H NMR (400 MHz, $CDCl_3$ + CCl_4; 1:1) spectrum of (2*S*,3a*R*,6*S*,7a*R*,8*S*)-(+)-2-(azidomethyl)-6,7,7-trimethylhexahydro-2*H*-3a,6methanobenzofuran-8-ol **16**.

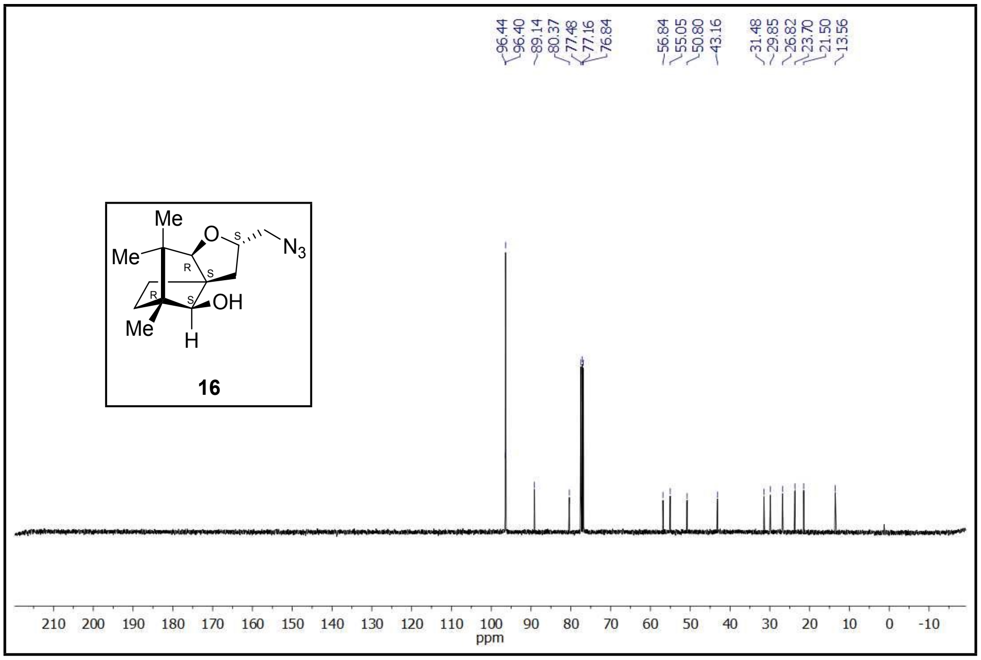

^{13}C NMR (100 MHz, $CDCl_3$ + CCl_4; 1:1) spectrum of (2*S*,3a*R*,6*S*,7a*R*,8*S*)-(+)-2-(azidomethyl)-6,7,7-trimethylhexahydro-2*H*-3a,6methanobenzofuran-8-ol **16**.

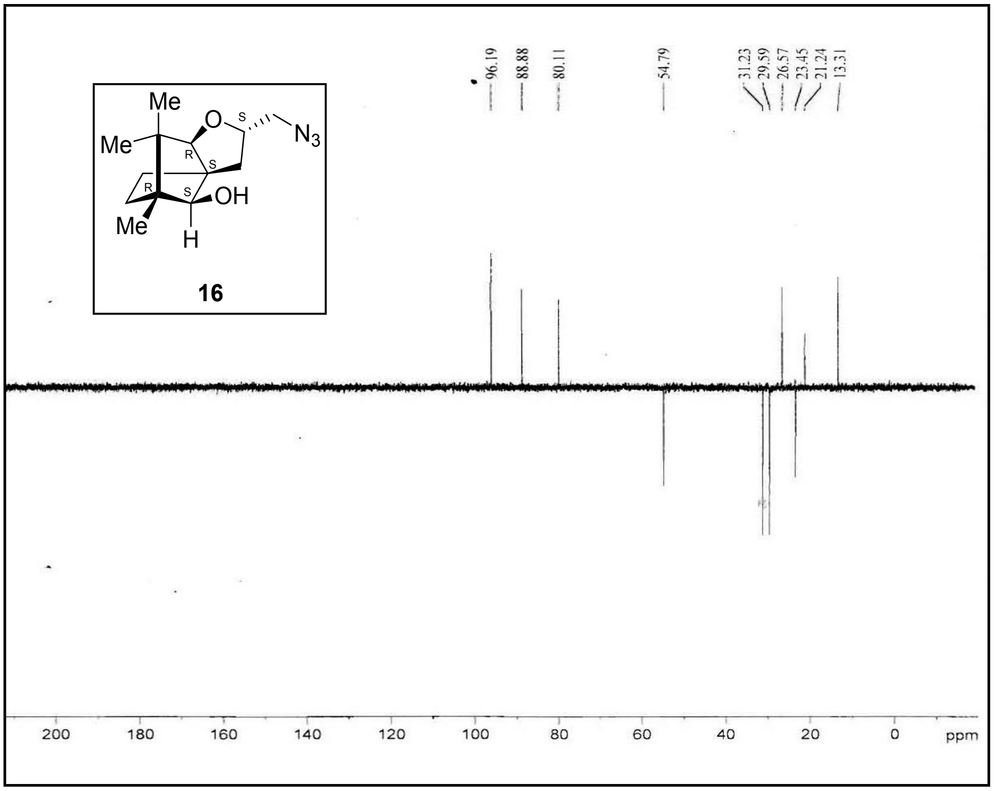

DEPT-135 NMR (100 MHz, $CDCl_3$ + CCl_4; 1:1) spectrum of (2*S*,3a*R*,6*S*,7a*R*,8*S*)-(+)-2-(azidomethyl)-6,7,7-trimethylhexahydro-2*H*-3a,6-methanobenzofuran-8-ol **16**.

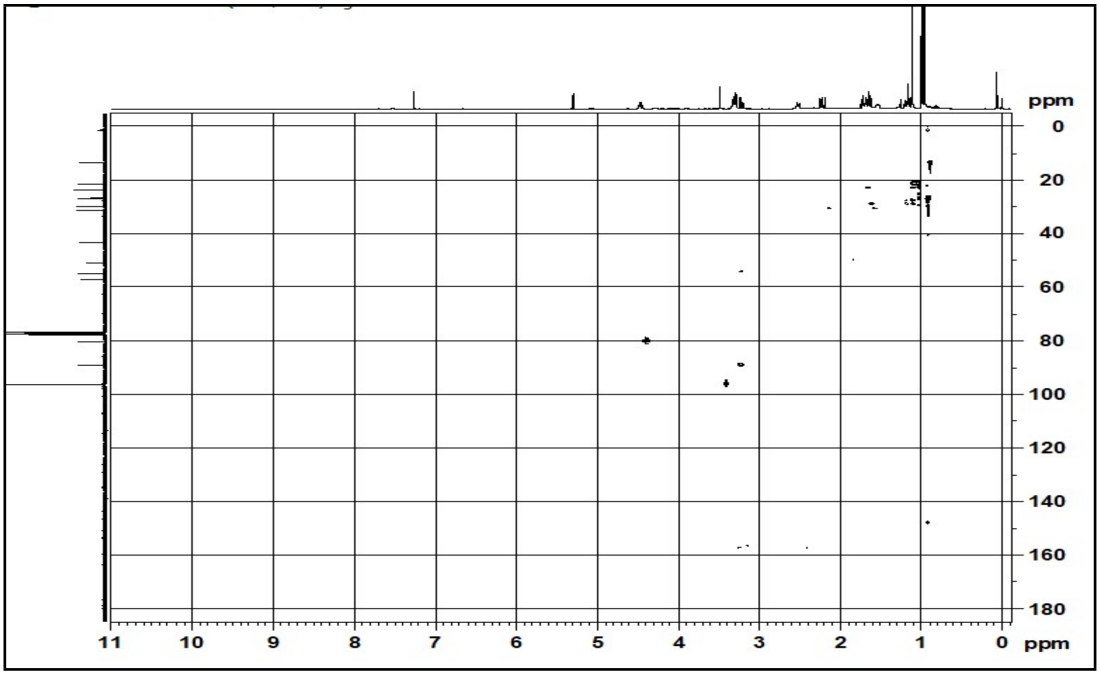

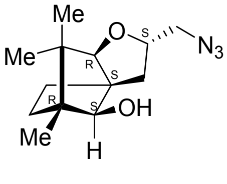

16

HSQC spectrum of (2*S*,3a*R*,6*S*,7a*R*,8*S*)-(+)-2-(azidomethyl)-6,7,7-trimethylhexahydro-2*H*-3a,6-methanobenzofuran-8-ol **16**.

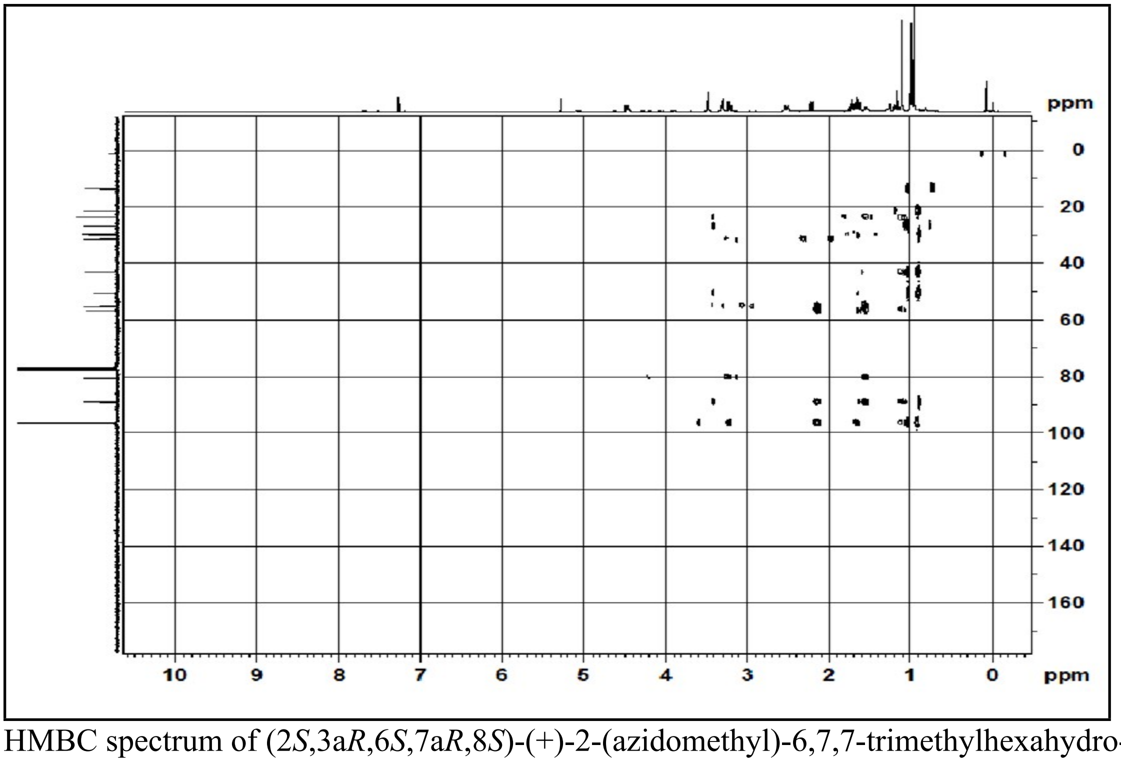

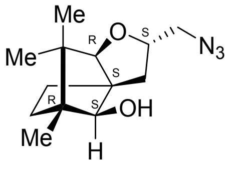

16

HMBC spectrum of (2*S*,3a*R*,6*S*,7a*R*,8*S*)-(+)-2-(azidomethyl)-6,7,7-trimethylhexahydro-2*H*-3a,6-methanobenzofuran-8-ol **16**.

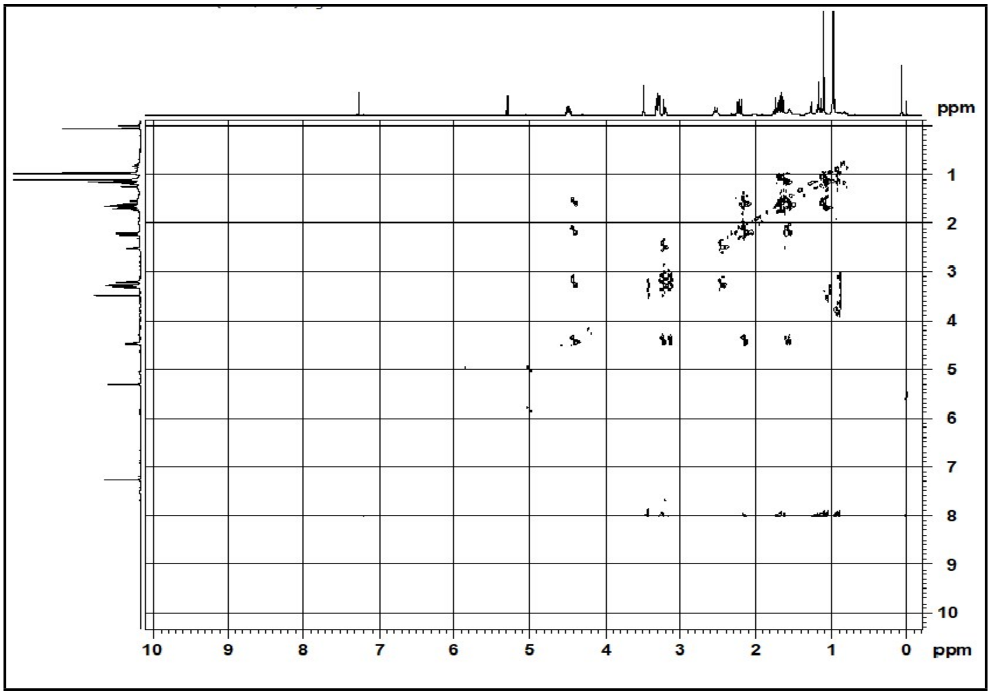

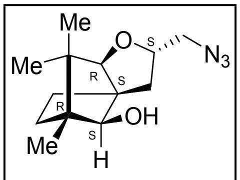

16

COSY spectrum of (2*S*,3a*R*,6*S*,7a*R*,8*S*)-(+)-2-(azidomethyl)-6,7,7-trimethylhexahydro-2*H*-3a,6-methanobenzofuran-8-ol **16**.

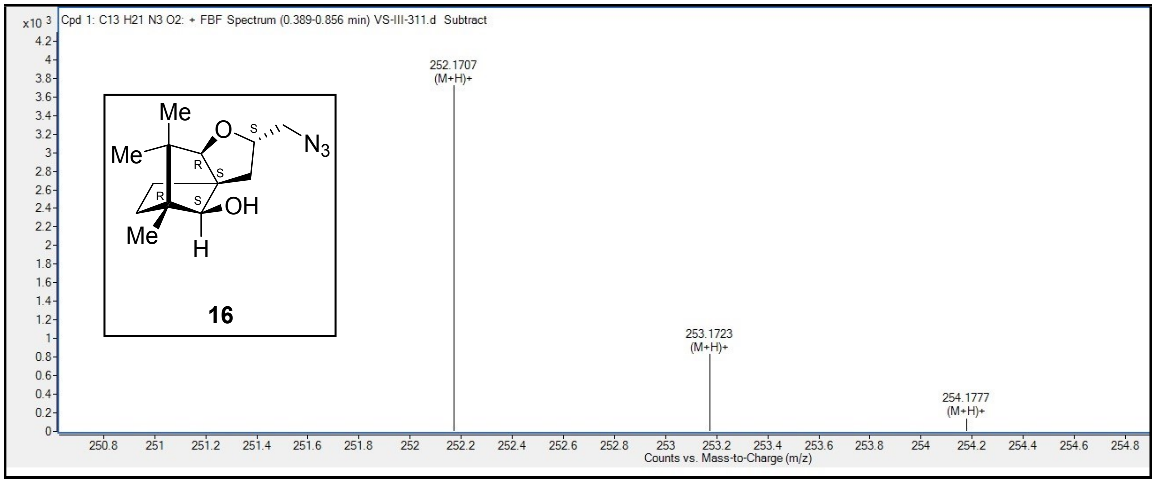

HRMS spectrum of (2*S*,3a*R*,6*S*,7a*R*,8*S*)-(+)-2-(azidomethyl)-6,7,7-trimethylhexahydro-2*H*-3a,6-methanobenzofuran-8-ol **16**.

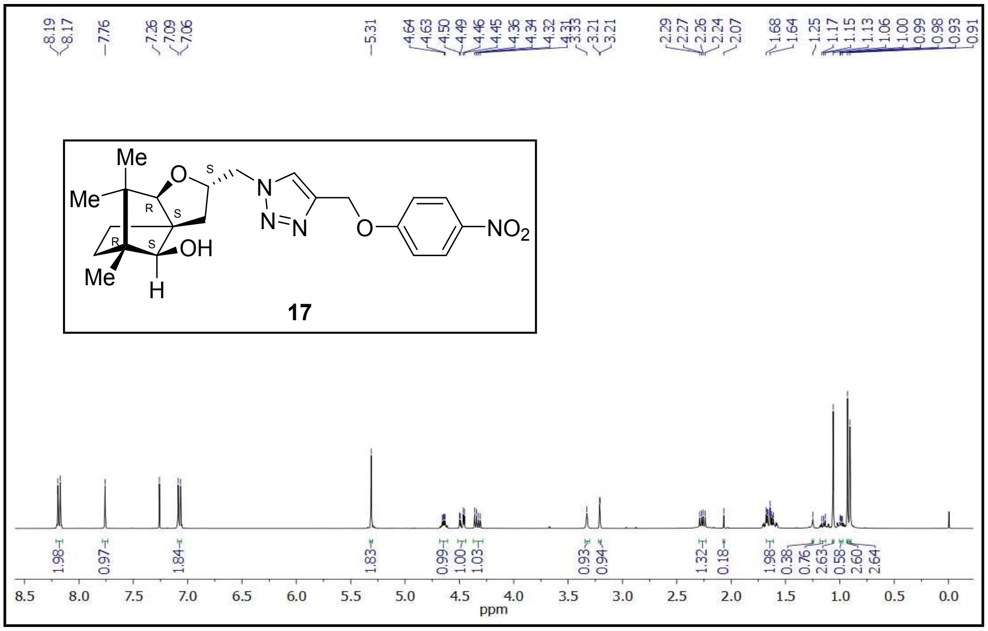

^{1}H NMR (400 MHz, $CDCl_3$ + CCl_4; 1:1) spectrum of (2*S*,3a*R*,6*S*,7a*R*,8*S*)-(+)-6,7,7-trimethyl-2-((4-((4nitrophenoxy)methyl)-1*H*-1,2,3-triazol-1yl)methyl)hexahydro-2*H*-3a,6-methanobenzofuran-8-ol **17**.

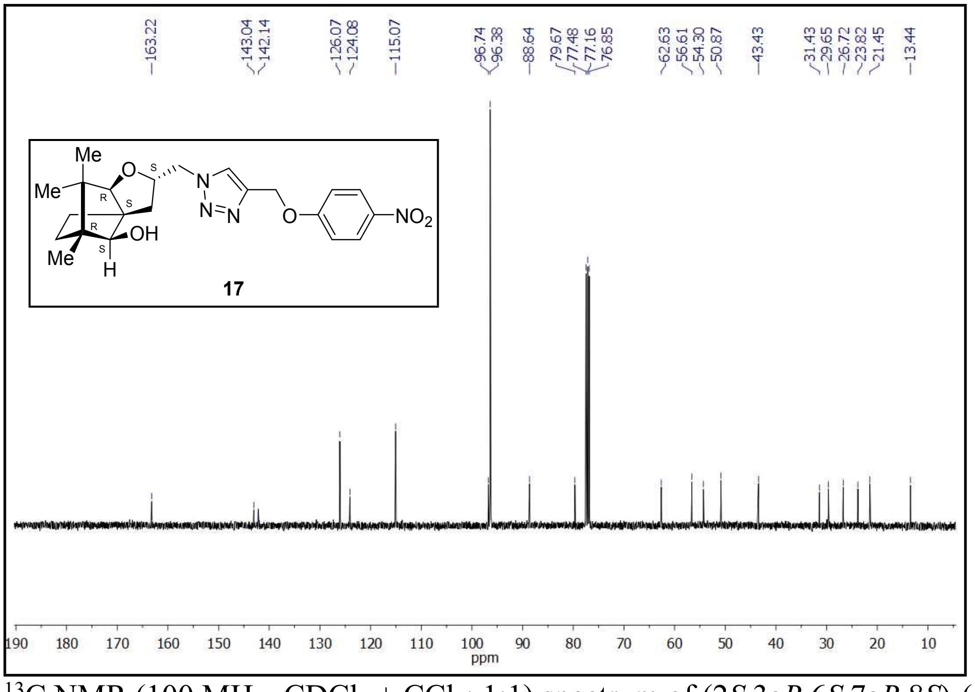

^{13}C NMR (100 MHz, $CDCl_3$ + CCl_4; 1:1) spectrum of (2*S*,3a*R*,6*S*,7a*R*,8*S*)-(+)-6,7,7-trimethyl-2-((4-((4nitrophenoxy)methyl)-1*H*-1,2,3-triazol-1yl)methyl)hexahydro-2*H*-3a,6-methanobenzofuran-8-ol **17**.

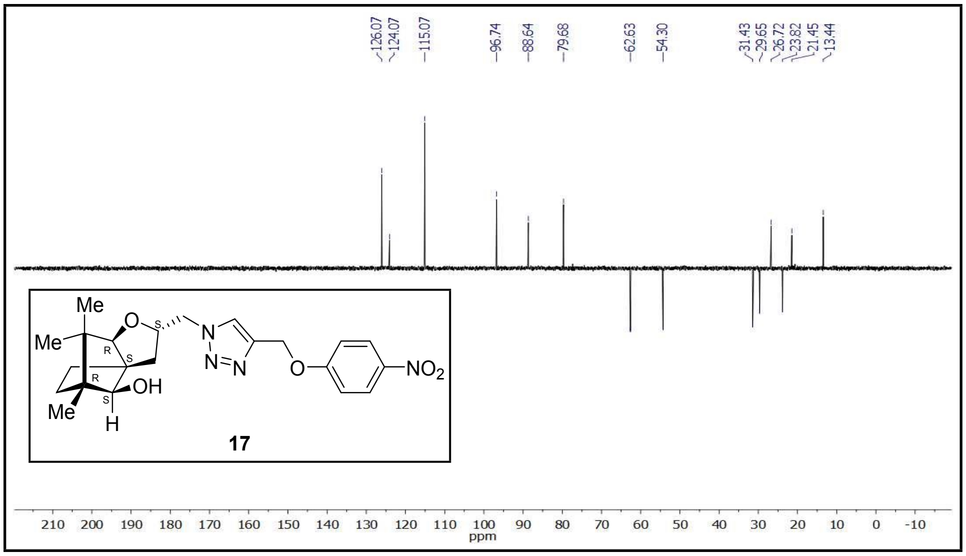

DEPT-135 NMR (100 MHz, $CDCl_3$ + CCl_4; 1:1) spectrum of (2*S*,3a*R*,6*S*,7a*R*,8*S*)-(+)-6,7,7-trimethyl-2-((4-((4nitrophenoxy)methyl)-1*H*-1,2,3-triazol-1yl)methyl)hexahydro-2*H*-3a,6-methanobenzofuran-8-ol **17**.

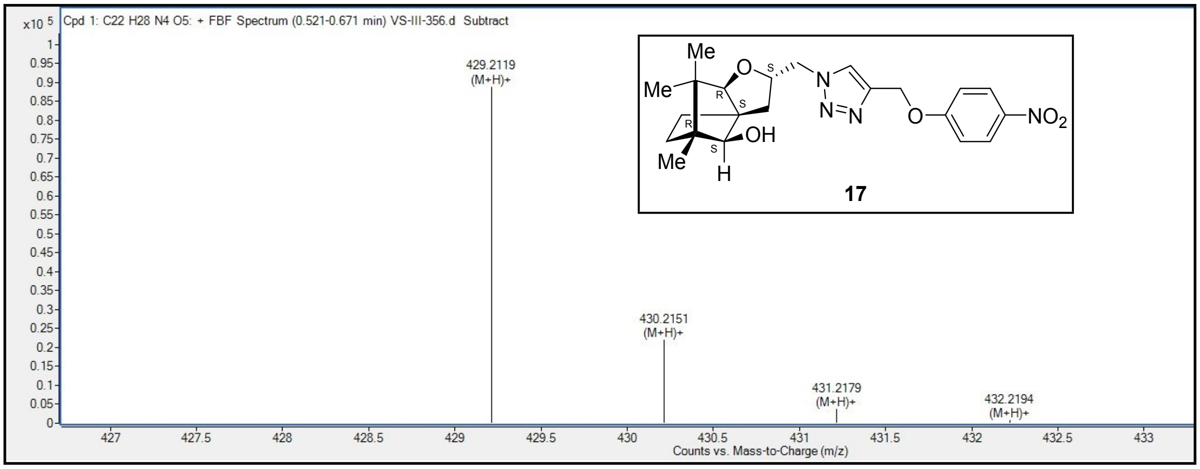

HRMS spectrum of (2*S*,3a*R*,6*S*,7a*R*,8*S*)-(+)-6,7,7-trimethyl-2-((4-((4nitrophenoxy)methyl)-1*H*-1,2,3-triazol-1yl)methyl)hexahydro-2*H*-3a,6-methanobenzofuran-8-ol **17**.
